# Clinical, Technical, and MRI Features Associated with Patients’ Outcome at 3 Months and 2 Years following Prostate Artery Embolization: Is There an Added Value of Radiomics?

**DOI:** 10.3390/jpm14010067

**Published:** 2024-01-04

**Authors:** Antoine Martin, Clément Marcelin, François Petitpierre, Eva Jambon, Rim Maaloum, Nicolas Grenier, Yann Le Bras, Amandine Crombé

**Affiliations:** 1 Department of Diagnostic and Interventional Radiology, Pellegrin University Hospital, Place Amélie-Raba-Léon, F-33076 Bordeaux, Francef.petitpierre@lecai.fr (F.P.); rim.maaloum@chu-bordeaux.fr (R.M.); nicolas.grenier@chu-bordeaux.fr (N.G.);; 2 BRIC Bordeaux Institute of Oncology, INSERM U1312, 2 Rue Dr Hoffmann Martinot, F-33000 Bordeaux, France; 3 Department of Radiology, Clinique Mutualiste de Pessac, 46 Avenue du Dr Albert Schweitzer, F-33600 Pessac, France

**Keywords:** prostate, prostate artery embolization, prognosis, outcome study, lower urinary tracts symptoms, magnetic resonance imaging, radiomics

## Abstract

Our aim was to investigate which features were associated with clinical successes at short- and mid-terms following prostate artery embolization (PAE) for symptomatic benign prostate hypertrophy (BPH). All adults treated by PAE for BPH at our referral center between January 2017 and March 2021, with pre-treatment MRI, technical success, and follow-up at 3 months and 2 years were included in this single-center retrospective study. Radiologists reviewed the prostatic protrusion index (PPI), adenomatous dominant BPH (adBPH), and Wasserman classification on pre-treatment MRI. Radiomics analysis was achieved on the transitional zone on pre-treatment T2-weighted imaging (WI) and ADC, and comprised reproducibility assessment, unsupervised classifications, and supervised radiomics scores obtained with cross-validated Elasticnet regressions. Eighty-eight patients were included (median age: 65 years), with 81.8% clinical successes at 3 months and 60.2% at 2 years. No feature was associated with success at 3 months, except the radiomics score trained on T2-WI and ADC (AUROC = 0.694). Regarding success at 2 years, no radiomics approaches provided significant performances; however, Wasserman type-1 and change in international prostate symptom score (IPSS) at 3 months ≤ −35% were associated with success in multivariable analysis (OR = 5.82, *p* = 0.0296, and OR = 9.04, *p* = 0.0002). Thus, while radiomics provided limited interest, Wasserman classification and early IPSS changes appeared predictive of mid-term outcomes.

## 1. Introduction

The therapeutic management of lower urinary tract syndrome (LUTS) due to benign prostate hypertrophy (BPH) is codified by national and international guidelines [1,2]. Transurethral resection of the prostate (TURP) is indicated after failure of medical treatments, however, its side effects, such as retrograde ejaculation (65%), erectile dysfunction (10%), or urinary incontinence (2%), can be problematic [3]. Therefore, minimally invasive techniques have emerged including prostate artery embolization (PAE), with increasing evidence regarding its efficiency, safety, and the possibility to re-perform PAE or TURP in case of symptoms recurrence [4,5].

Indeed, a randomized trial has demonstrated fewer adverse events after PAE compared to TURP, but a slightly lower decrease in international prostate symptom score (IPSS) at 24 months [6]. In 1072 patients followed over 10 years, Bilhim et al. showed that PAE provided long-term LUTS relief, with re-intervention rates of 20% within 5 years [5]. It seems that the prostatic tissue could re-grow in 50% of patients after PAE, within 5 years, leading to the re-appearance of LUTS [5]. Overall, these findings stress the need to better select patients who may benefit from PAE.

Thus, several studies have investigated features associated with PAE success but with diverging results. High prostate volume, younger age, and adenomatous benign BPH (adBPH) have been linked with higher rates of success [7,8,9,10] whereas cardiovascular comorbidities, unilateral embolization, or glue and large microspheres may provide worse prognosis [8,11,12]. Moreover, radiological features assessing the shape of the adenoma such as prostate protrusion index (PPI) within the bladder, or the presence of a median lobe have shown controversial results [4,10,13,14]. Lastly, Boschheidgen et al. have suggested that a simplified Wasserman classification for BPH would correlate with PAE success [10].

Overall, these studies suggest an impact of the nature of the adenoma itself, in terms of textural patterns and shape on the PAE efficacy. Yet, the radiological analyses of the BPH remained mostly based on qualitative assessments. Radiomics features (RFs) have emerged as innovative numeric features able to extensively quantify the rearrangements of gray-level voxels on any imaging modality [15,16]. Those RFs can help identify relationships between radiological phenotypes and patients’ outcomes through supervised analyses relying on machine-learning algorithms or to discover hidden imaging patterns using unsupervised clustering [15,16].

Thus, we hypothesized that RFs could deepen the analysis of BPH patterns on pre-PAE MRI and subsequently correlate with PAE clinical efficacy in short term (i.e., 3 months) and mid-term (i.e., 2 years), independently of clinical, technical, and conventional radiological characteristics.

## 2. Materials and Methods

### 2.1. Study Design

This single-center retrospective observational study was approved by the institutional review board of Bordeaux University Hospital (CER-BDX-2023-107). Written consent was waived by its retrospective nature.

All consecutive adults treated by PAE for BPH because of LUTS between January 2017 and March 2021 at our tertiary interventional radiology units were included as they filled the following inclusion criteria: available pre-PAE MRI including axial T2-weighted imaging (WI) of good quality, contraindication or refusal for TURP, pre-treatment IPSS (IPSS_0_) ≥ 8, prostatic volume ≥ 35 mL, bilateral PAE, available IPSS at 3 months (IPSS_M3_), and patients’ satisfaction at 3 months and 2 years.

Exclusion criteria were suspected prostate cancer based on prostate-specific antigen (PSA) and MRI, medical history of prostate cancer, prostatic surgery, or prostate embolization for any reason, indwelling bladder catheter, and age < 50 years.

**Figure 1:**
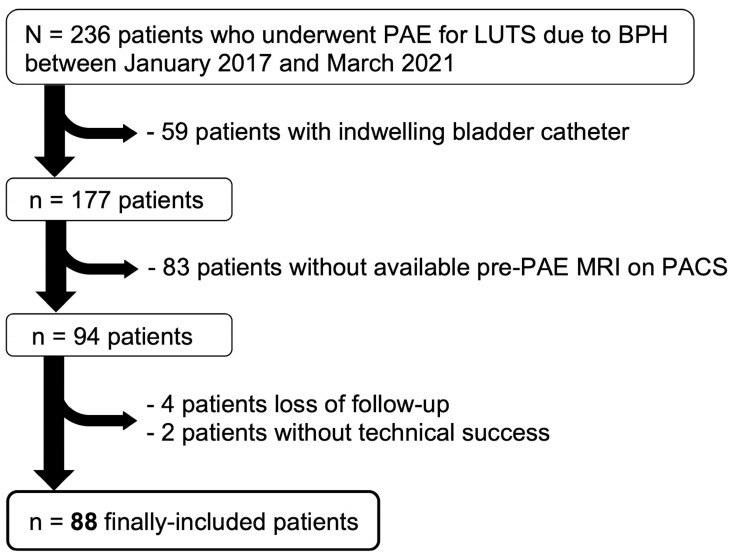
Study flow-chart. Abbreviations: BPH: benign prostate hyperplasia, LUTS: lower urinary tract symptoms, PACS: picture archiving and communication system, PAE: prostate artery embolization.

Figure 1 shows the flow chart.

The following clinical data were collected from medical reports: age (categorized as <65 years, 65–74 years, and ≥75 years), body mass index (BMI in kg·m^−2^, categorized as <25, 25–29 and ≥30), cardiovascular risk factors (categorized as diabetes and/or past cardiovascular event versus none), IPSS_0_ and quality of life (QOL_0_) scoring.

### 2.2. MRI_0_ Acquisitions

Pre-treatment MRIs (MRI_0_) were acquired on MR systems at our center (1.5-T Ambition Blueseal, Philips, Best, The Netherland, and 3-T MR-750W, General Electrics, Milwaukee, WI, USA) and outside center (1.5-T Signa Artist, General Electrics, Milwaukee, WI, USA; 1.5-T Ingenia, Philips, Best, The Netherland). Abdominal surface arrays were used without endorectal coils. All protocols comprised, at least, one axial and one sagittal turbo spin echo T2-WI (TE/TR = 70–192/3442–6135 msec, matrix = 220 × 220–288 × 288, field-of-view = 200–240 × 178–240, in-plane resolution = 0.6 × 0.6–0.8 × 0.8 mm^2^, slice thickness = 3 mm), and, not systematically, axial diffusion-weighted imaging (DWI, with the following b-values: 0, 100 and 1000 s·mm^−2^, TE/TR = 60–200/3400–4000 msec, matrix = 80–120/80–120, field-of-view = 160 × 160, in-plane resolution = 1.6–2.6 × 1.6–2.6 mm^2^, slice thickness = 4–4.7 mm). Apparent diffusion coefficient (ADC) maps were reconstructed using a mono-exponential decay model.

### 2.3. Radiological Analysis

Three radiologists (C.M. and E.J., senior radiologists with 10 and 8 years of experience in urological imaging, respectively, and A.M. a fellow with a 6-month internship in interventional radiology and urological imaging) double-blinded assessed the following radiological features from MRI_0_:

(i) PPI, categorized as: grade 1 + 2: ≤10 mm and grade 3: >10 mm [17];
(ii) adBPH according to Little et al. (defined as two or more adenomas [i.e., with intermediate T1-WI/T2-WI signal, surrounded by a low T1-WI/T2-WI capsule and marked contrast-enhancement after gadolinium-chelates injection] within the peri-urethral transition zone of 1 cm or greater) [9];
(iii) qualitative assessment of the signal intensity (SI) of the transitional zone on T2-WI (subjectively categorized as high, intermediate, or low compared to the pelvic muscles);
(iv) simplified Wasserman classification (categorized as type 1: bilateral transitional zone hyperplasia, type 2: retro-urethral median lobe hyperplasia [reference], type 3: type 1 + type 2) [10,18];
(v) intra-bladder lithiasis;
(vi) bladder diverticula.

Those three readings enabled to evaluate the inter-observer reproducibility. The reading from the most experienced radiologist (C.M.) was used for the remaining statistical analyses.

### 2.4. Radiomics Analysis

The principle of radiomics approaches is to provide an extensive quantification of the radiological phenotypes (or radiophenotypes) of objects of interest (herein, the 3D segmentation of the prostate) on any medical imaging using mathematical operators that enable to assess the texture and shape through the extraction of several radiomics features [RFs] [15,16]. The commonest and most standardized RFs are based on histogram analysis of the various gray levels contained in the segmentation, on texture analysis of the 3D re-arrangements of those gray levels using gray-level matrices, and lastly by applying shape analysis. After extracting hundreds of RFs, the next step is to explore their relationship to relevant clinical outcomes, such as clinical success after a therapeutic procedure. This data-mining step is usually achieved through supervised analyses in which the outcome to predict is clearly identified and labeled in radiomics databases, using machine-learning algorithms trained in cross-validation. Alternatively, unsupervised analyses can be investigated, which consists of performing clustering of the observations based on their RFs without any *a priori*, and secondarily understanding the unsupervised patterns of patients.

**Figure 2:**
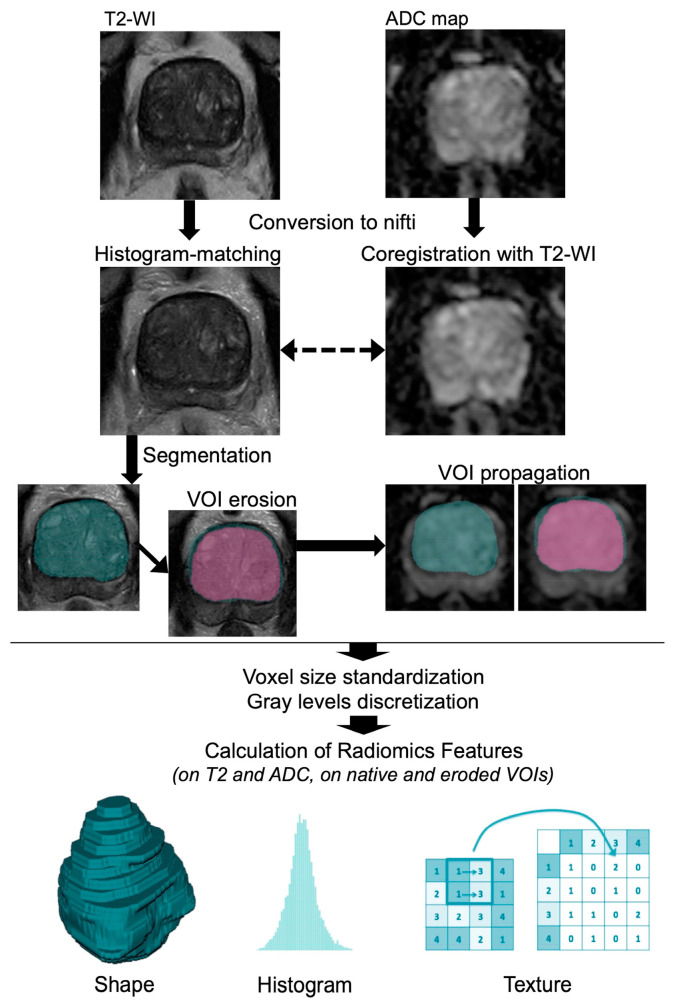
Radiomics workflow performed on pre-treatment MRI. Abbreviations: ADC: apparent diffusion coefficient, VOI: volume of interest, WI: weighted imaging.

Figure 2 illustrates the radiomics workflow of this study.

#### 2.4.1. Radiomics Processing

The radiomics analysis was achieved on the axial T2-WI and axial ADC map from MRI_0_. As T2-WI sequences are not standardized by nature, the intensities on T2-WI dataset were homogenized from 0 to 1000, which was achieved with R (v. 4.1.0, Vienna, Austria) by using histogram-matching with 1000 landmarks (‘hatch’ package, github.com/abdhigithub/hatch, accessed on 1 July 2023), after converting MRIs to the nifti format (‘dcm2niir’ package, github.com/muschellij2/dcm2niir, accessed on 1 July 2023). Since ADC values are measured with a standardized unit (mm·s^−2^), the ADC map was not homogenized, however, it was co-registered to the T2-WI using a rigid registration (‘ANTsR’ package, github.com/ANTsX/ANTsR, accessed on 1 July 2023). We purposely decided not to use a denoising algorithm in order not to alter the texture of the prostate segmentation.

Afterward, the post-processed T2-WI and ADC sequences were exported to the LIFEx freeware (version 7.1.17, https://www.lifexsoft.org/, accessed on 1 July 2023) [19], which is compliant with the international biomarker standardization initiative (IBSI, https://theibsi.github.io/, accessed on 1 July 2023). One radiologist (A.M.) manually segmented in 3D, slice-by-slice, the transitional zone of each prostate on the T2-WI, then propagated the corresponding volume-of-interest (VOI) on the ADC and manually adjusted the boundaries if needed (which could happen despite co-registration because of geometric distortion with DWI), providing the VOI_T2_ and VOI_ADC_. A senior radiologist (A.C. with 6 years of experience in oncologic and urological imaging) verified all the VOIs and adjusted them if needed, as an additional quality control.

Additionally, the two radiologists also measured the volume of the peripheral zone of the prostate to calculate the ratio between transitional zone and peripheral zone volumes (rTZPZ).

#### 2.4.2. Features Extraction

Beforehand, voxel sizes were homogenized to a common resolution of 1 × 1 × 4 mm^3^ for T2-WI and 2 × 2 × 5 mm^3^ for ADC, thanks to b-spline interpolator. Gray levels were discretized to 128 units between SI = 0 and 1000 for T2-WI and between ADC = 0 and 3 mm^2^·s^−1^ for ADC map. Overall, 156 texture RFs were extracted from T2-WI and ADC (52 from histogram-based features, 72 from gray-level co-occurrence matrices [GLCM, with 1, 2, and 4 voxel displacements], 5 from neighborhood gray-tone difference matrix [NGTDM], 11 from gray-level run-length matrix [GLRLM] and 16 from gray-level size zone matrix [GLSZM]). Additionally, a total of 14 Shape RFs were extracted from T2-WI because it was the most anatomical imaging. Thus, we extracted a total of 326 RFs, whose definitions are detailed on the LIFEx website (https://www.lifexsoft.org/, accessed on 1 July 2023).

The average and standard deviation of the ADC value of the transitional zone were studied individually as they can be easily calculated on clinical PACS.

### 2.5. PAE Procedure

PAE was performed by senior interventional radiologists (F.P. and C.M., with 8 and 5 years of experience, respectively) following guidelines [20], and under local anesthesia. No bladder catheterization, non-steroid anti-inflammatory drugs, or any other medication was required before, during, or after PAE. An Artis Pheno (Siemens, Erlangen, Germany) angiography suite was used for all patients. Briefly, the steps were: (i) insertion of a 5Fr vascular sheath in the right common femoral artery or in the left radial artery; (ii) contralateral internal iliac artery (IIA) cannulation, using UAC catheter (Impress^®^ UAC2 Merit Medical, South Jordan, UT, USA) for femoral access procedure and HH catheter (Impress^®^ HH Merit Medical, South Jordan, UT, USA) for radial access; (iii) a single 3D rotational cone-beam CT angiography; (iv) superselective catheterization of prostatic arteries with the microcatheter located in the middle third of the prostatic artery using a 2.0 Fr microcatheter (Progreat^®^ Micro Catheter System, Terumo, Tokyo, Japan). If possible, a single microcathether was used for both sides after cleaning with G5% solution.

In case of risk of non-target embolization due to anastomotic vessel, a protective coil embolization was performed. The embolization agents were categorized as microspheres, glues (N-butyl cyanoacrylate-based), or microspheres + glues. Regarding microspheres, the embolization was performed using 1 mL of 100–300 μm or 300–500 μm microspheres (Embogold^®^, Merit Medical, South Jordan, Utah) mixed with saline and contrast agent up to 20 mL (Visipaque 270, GE Healthcare, Chicago, IL, USA). The injected volume ranged from 4 to 30 mL depending on the vascular flow. Regarding glues, NBCA (Glubran 2^®^, GEM, Italy) was diluted with iodized oil (Lipiodol, Guerbet, France) to make it visible during fluoroscopy and to improve its fluidity (mostly 1/9 [range of dilution ratio: 1/7–1/11] as the best compromise between distal embolization and low risk of reflux). The injected volume ranged from 0.3 to 0.6 mL depending on the vascular flow. The injection was stopped when a significant reflux occurred over the first 1–2 mm at the extremity of the microcatheter. Vascular access was secured using occlusion devices (TR band [Terumo^®^, Tokyo, Japan] for radial access, and Exoseal [Cordis^®^, Santa Clara, CA, USA] for rCFA access). Patients were monitored in the interventional unit for the first hour and in the ambulatory surgery department for 2 to 6 h depending on the radial or femoral access. Immediate complications were recorded. If the procedure could not be bilateral on the first attempts, a complementary procedure was performed within 6 weeks, hence, all embolizations were ultimately bilateral. Ultimately, all included PAEs were considered technically successful by the interventional radiologist who performed them.

### 2.6. Patients’ Follow-Up and Outcomes

#### 2.6.1. Short-Term Outcomes

Post-PAE consultation and imaging (mainly MRI [MRI1], alternatively ultrasonography) were systematically planned 3 months after the procedure, which enabled the collection of the IPSS_M3_, QOL_M3_, patients’ satisfaction, and delayed complications, and subsequently the absolute and relative change in IPSS and QOL. When MRI_1_ was available, one radiologist (A.M.) measured the post-PAE prostate volume to calculate the absolute and relative changes in prostate volume. No radiomics or other radiological features were extracted from MRI_1_. We defined short-term clinical success as an absolute decrease in IPSS ≤ −4 and a patient clinically satisfied with PAE.

#### 2.6.2. Mid-Term Outcomes

Lastly, the post-PAE medical records over at least 2 years after PAE were analyzed for all patients and one radiologist contacted them all during the 1st trimester of 2023. Clinical success at mid-term was defined as patient satisfaction at 2 years without the need for new PAE or prostate surgery.

### 2.7. Statistical Analyses

**Figure 3:**
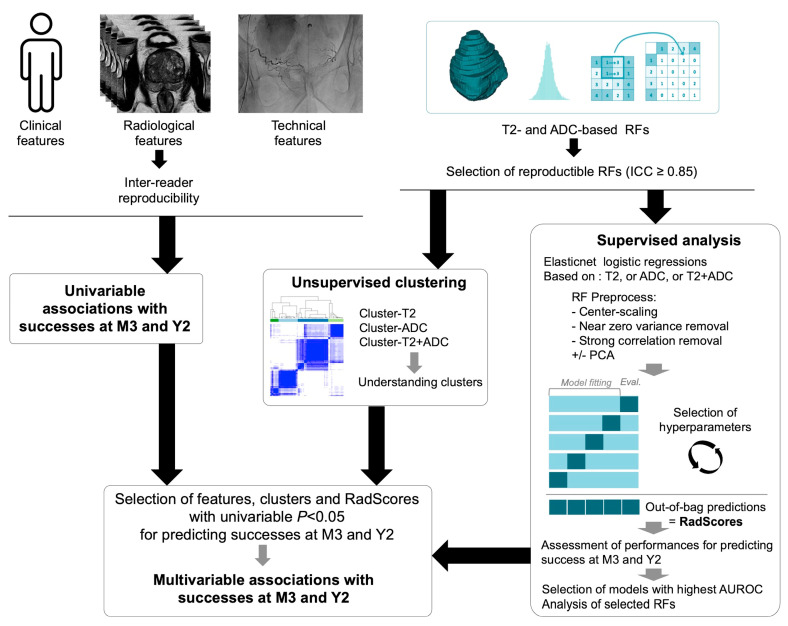
Statistical pipeline. Clinical, radiological, and radiomics were all obtained before prostate artery embolization (PAE). Technical features were obtained during PAE. Abbreviations: ADC: apparent diffusion coefficient, AUROC: area under the ROC curve, ICC: intra-class correlation coefficient, PCA: principal component analysis, M3: at 3 months after prostate artery embolization, RF: radiomics feature, Y2: at 2 years after prostate artery embolization.

Statistical analyses were also performed with R. All tests were two-tailed. A *p*-value < 0.05 was deemed significant. The statistical pipeline is represented in Figure 3.

#### 2.7.1. Reproducibility of Radiological and Radiomics Features

The inter-rater reproducibility of the non-ordinal and ordinal categorical radiological features over the 3 readers was assessed with Cohen’s Kappa (κ), and Krippendorf’s alpha (α), respectively.

We excluded non-reproducible RFs across slight perturbations of the segmented VOIs. To do so, we automatically eroded VOI_T2_ and VOI_ADC_ by one voxel using LIFEx, and we re-extracted similarly the RFs. Hence, for each RF, we obtained paired values (for the initial VOI and the eroded VOI), which enabled us to calculate the intra-class correlation coefficient (ICC) of each RF and to only select reproducible RFs, i.e., with ICC ≥ 0.85 (‘irr’ package, github.com/cran/irr, accessed on 1 July 2023). This method is an alternative to the re-iteration of the whole segmentation process, which is time-consuming and costly for medical resources.

#### 2.7.2. Associations with Short- and Mid-Term Outcomes

Univariable associations between numeric explanatory variables and outcomes were tested with unpaired Student *t*-test or Mann-Whitney Wilcoxon test depending on the Shapiro-Wilk normality test. For numeric variables associated with clinical successes, ROC curves were plotted and optimal cut-offs according to the Youden index were determined [21]. Univariable associations between categorical explanatory variables and outcomes were investigated with Fisher or Chi-square tests, as appropriate. Odds ratios (ORs) with 95% confidence intervals (CIs) were estimated with univariable logistic regression.

#### 2.7.3. Radiomics Analyses

Herein, we aimed at investigating the meaning of pre-PAE radiomics of the prostate and whether prostate radiophenotypes were associated with good short- and long-term clinical outcomes.

First, we investigated the univariable associations. Benjamini-Hochberg correction was applied to adjust for multiple comparisons.

Secondly, we developed unsupervised classifications of the prostates based on pre-treatment T2-WI (cluster-T2), on ADC (cluster-ADC), and both (cluster-ADC + T2). After center-scaling the corresponding RFs, a consensual hierarchical clustering was performed using the Person distance and the average link; each clustering was resampled 10,000 times by leave-one-out of 30% of the samples [22]. The best number of groups per cluster was identified using the consensus cumulative distribution function and the delta area plot [22]. The univariable associations of cluster-T2, cluster-ADC, cluster-ADC+T2 with initial prostate volume, IPSS and QOL, adBPH, Wasserman classification, and clinical success at 3 months and 2 years were investigated (with adjustments for multiple comparisons using Benjamini-Hochberg procedure).

Thirdly, we developed a supervised radiomics score to predict both outcomes using either T2-based RFs, ADC-based RFs, or T2 + ADC-based RFs. We trained Elasticnet penalized logistic regression (elasticnet-LR) in 5-fold cross-validation [23,24]. Elasticnet regression is a supervised method that enables to perform variable selection and regularization in the setting of highly multidimensional datasets (such as radiomics datasets) where the number of variables strongly exceeds the number of observations. It combines two regularization methods:

- LASSO regularization (for least absolute shrinkage and selection operator), which minimizes the usual sum of squared errors thanks to a bound of the absolute values of the coefficients or penalty function.
- Ridge regularization, which is another penalization method that adds a quadratic part to the penalty function.

Therefore, there are two hyperparameters to tune in Elasticnet before training one algorithm: (i) α, which controls the Elasticnet penalty and ranges from 0 (complete ridge regression) and 1 (complete LASSO regression), and (ii) λ, which controls the overall strength of the penalty. A grid search was provided to the Elasticnet algorithm before it was trained in 5-fold cross-validation, namely: (i) α: from 0 to 1 with 0.025 increments (i.e., 0, 0.025, 0.050, …, 1), and (ii) λ: from 0 to 5 with 0.2 increments (i.e., 0, 0.2, 0.4, …, 5).

The reproducible RFs entered in the modeling were pre-processed within the cross-validation as follows: (i) center-scaling, (ii) near-zero-variance removal, (iii) highly correlated features removal (>0.75), and (iv) dimensionality reduction using principal component analysis (PCA). The area under the ROC curve (AUROC) with 95%CI was estimated on the unseen out-of-bag data of the cross-validation schemes and was defined as the main performance measure to identify the best elasticnet-LR model. Next, we extracted the selected RFs, their coefficients, and the out-of-bag predictions for each patient (named: RadScore-T2M3, RadScore-ADCM3, RadScore-T2.ADCM3 for clinical success at 3 months, and RadScore-T2Y2, RadScore-ADCY2, RadScore-T2.ADCY2 for clinical success at 2 years) and tested the associations with both outcomes. ROC comparisons were achieved with Delong tests [25].

#### 2.7.4. Final Multivariable Analyses

All variables (clinical, radiological, technical, clusters, and radiomics scores) with a univariable *p*-value < 0.05 were entered in multivariable logistic regression to estimate multivariable ORs with 95%CI and subsequently independent predictors for both outcomes. The age groups and embolization material were added to the modeling as potentially confounding covariables.

## 3. Results

### 3.1. Patients’ Characteristics

Eighty-eight patients were finally included (Figure 1, Table 1). The median age was 65 years (IQR: 62–70, range: 54–84).

**Figure 4:**
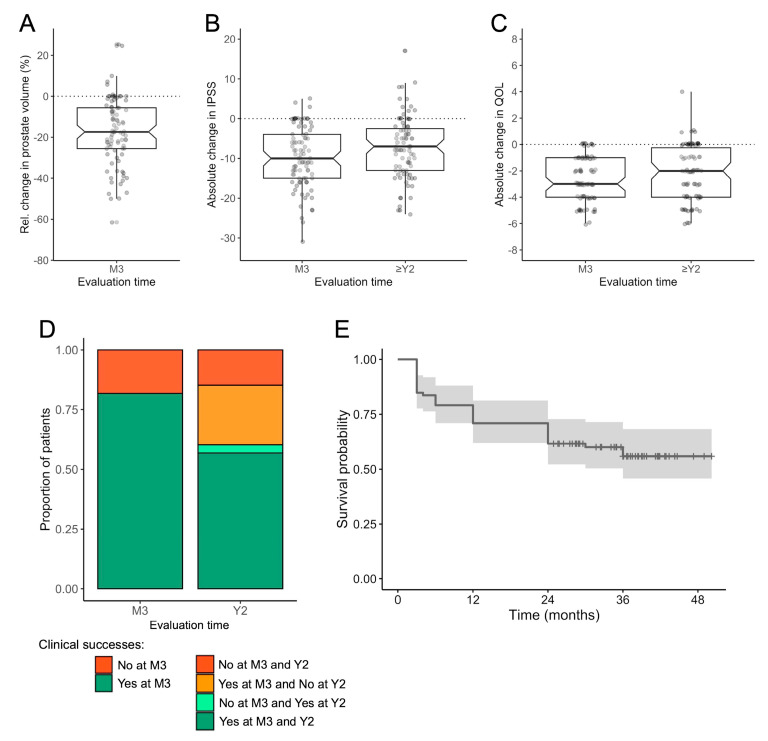
Patients’ outcomes following prostate artery embolization (PAE) for benign prostate hypertrophy. (**A**) Relative change in prostate volume between the pre-PAE MRI and the revaluation MRI at 3 months (M3) post-PAE. (**B**) Absolute changes in IPSS at M3 and the latest evaluation (after at least two years [Y2] of follow-up). (**C**) Absolute changes in QOL at M3 and the latest evaluation. (**A**–**C**) are boxplots with median, 1st, and 3rd quartiles with all patients being represented with points. (**D**) Proportion of patients with clinical success at M3 and Y2. (**E**) Kaplan-Meier.

Figure 4 represents the main outcomes. The average absolute decrease in IPSS was −9.8 ± 7.4 at 3 months after PAE and −7.3 ± 7.8 at the last follow-up between 2 and 5 years post-PAE (−11.5 ± 5.2 in those who did not relapse).

The average absolute decrease in QOL was −2.6 ± 1.6 at 3 months and −2.2 ± 2 at the last follow-up (−3.3 ± 1.7 in those who did not relapse). The average relative change in prostate volume at 3 months was −17 ± 16% (Supplementary Table S1).

There were 72/88 (81.8%) clinical successes at 3 months and 53/88 (60.2%) clinical successes at 2 years. There were 3/88 (3.4%) patients who did not reach clinical success at 3 months but later. Conversely, there were 22/88 (25%) patients who had clinical success at 3 months but declared a relapse during the follow-up.

Regarding PAE complications, 11/88 (13.6%) declared acute minor complications (i.e., post-embolization syndrome), 2/88 (1.1%) acute major complication (i.e., glans ulceration or necrosis), and 1/88 (1.1%) both acute minor and major complications (i.e., thrombosis of the radial artery). None presented delayed complications.

Regarding patients whose symptoms relapsed after PAE, 11 underwent prostate surgery.

### 3.2. Univariable Analysis of Clinical, Radiological, and Technical Characteristics (Table 2)

No characteristic was associated with clinical success at 3 months.

Regarding clinical success at 2 years, the following variables were associated: the Wasserman classification (*p* = 0.0447, with higher odds for clinical success for Wasserman type 1, OR = 6.67, 95%CI = 1.76–29.76, compared to type 2), absolute and relative change in IPSS at 3 months (with higher odds for clinical success in case of higher IPSS decrease, OR = 0.91, 95%CI = 0.85–0.97, and OR = 0.97, 95%CI = 0.95–0.98, respectively), and absolute and relative change in QOL at 3 months (with higher odds for clinical success in case of higher QOL decrease, OR = 0.55, 95%CI = 0.38–0.75 and OR = 0.97, 95%CI = 0.95–0.98, respectively).

**Table 2:** Assessment of the univariable associations between clinical, radiological, and technical variables and clinical successes at 3 months and 2 years after PAE.

Characteristics		Clinical Success at 3 Months			Clinical Success at 2 Years		
		No	Yes	*p*-Value	No	Yes	*p*-Value
Pre-PAE clinical variables							
Age groups				0.2723			0.1866
	≥75 years	2/16 (12.5)	9/72 (12.5)		4/35 (11.4)	7/53 (13.2)	
	65–74 years	4/16 (25)	33/72 (45.8)		11/35 (31.4)	26/53 (49.1)	
	<65 years	10/16 (62.5)	30/72 (41.7)		20/35 (57.1)	20/53 (37.7)	
BMI groups				0.8128			0.11367
	Obese (≥30 kg/m^2^)	1/13 (7.7)	10/70 (14.3)		3/31 (9.7)	8/52 (15.4)	
	Overweight (25–29 kg/m^2^)	6/13 (46.2)	30/70 (42.9)		10/31 (32.3)	26/52 (50)	
	Normal (<25 kg/m^2^)	6/13 (46.2)	30/70 (42.9)		18/31 (58.1)	18/52 (34.6)	
Medical treatment for BPH				0.6257			>0.9999
	Yes	13/16 (81.2)	50/70 (71.4)		26/35 (74.3)	37/51 (72.5)	
	No	3/16 (18.8)	20/70 (28.6)		9/35 (25.7)	14/51 (27.5)	
Cardiovascular comorbidities				0.3025			0.8830
	Yes	8/14 (57.1)	27/71 (38)		14/32 (43.8)	21/53 (39.6)	
	No	6/14 (42.9)	44/71 (62)		18/32 (56.2)	32/53 (60.4)	
Pre-PAE IPSS		17.8 ± 6.1	18.9 ± 6.3	0.5073	19.6 ± 6.4	18.1 ± 6.2	0.2551
Pre-PAE QOL		4.9 ± 1.4	4.7 ± 1.0	0.1493	4.9 ± 0.9	4.6 ± 1.1	0.3356
Pre-PAE radiological variables							
Bladder diverticulum				0.6761			0.2169
	Yes	1/16 (6.2)	10/72 (13.9)		2/35 (5.7)	9/53 (17)	
	No	15/16 (93.8)	62/72 (86.1)		33/35 (94.3)	44/53 (83)	
Simplified Wasserman classification				0.5026			0.0181 *
	Type 1	3/16 (18.8)	11/72 (15.3)		10/35 (28.6)	4/53 (7.5)	
	Type 2	4/16 (25)	29/72 (40.3)		9/35 (25.7)	24/53 (45.3)	
	Type 3	9/16 (56.2)	32/72 (44.4)		16/35 (45.7)	25/53 (47.2)	
Signal intensity on T2-WI				>0.9999			0.2193
	Low or intermediate	7/16 (43.8)	30/72 (41.7)		18/35 (51.4)	19/53 (35.8)	
	High	9/16 (56.2)	42/72 (58.3)		17/35 (48.6)	34/53 (64.2)	
Prostatic protrusion index				0.4543			0.3764
	I or II	8/16 (50)	46/72 (63.9)		19/35 (54.3)	35/53 (66)	
	III	8/16 (50)	26/72 (36.1)		16/35 (45.7)	18/53 (34)	
adPBH				0.8987			>0.9999
	Yes	10/16 (62.5)	41/72 (56.9)		20/35 (57.1)	31/53 (58.5)	
	No	6/16 (37.5)	31/72 (43.1)		15/35 (42.9)	22/53 (41.5)	
Bladder lithiasis				>0.9999			>0.9999
	Yes	0/16 (0)	1/72 (1.4)		33/35 (94.3)	44/53 (83)	
	No	16/16 (100)	71/72 (98.6)		2/35 (5.7)	9/53 (17)	
Prostate volume (mL)		92.5 ± 39.7	92.1 ± 41.2	0.8202	87.1 ± 38.6	95.5 ± 42	0.4302
Ratio TZ/PZ		8.4 ± 7.5	9.3 ± 7.7	0.5554	895.7 ± 649.3	931.7 ± 834	0.7331
Mean ADC (mm^2^/s)		1.018 ± 0.429	1.093 ± 0.454	0.3898	1091.4 ± 413.7	1070.1 ± 473.4	0.9326
SD ADC (mm^2^/s)		0.175 ± 0.106	0.178 ± 0.086	0.6439	192.1 ± 94	168.1 ± 86.5	0.2239
Per-PAE technical variables							
Embolization material				0.2323			0.3148
	NBCA	13/16 (81.2)	43/72 (59.7)		22/35 (62.9)	34/53 (64.2)	
	Microparticles	1/16 (6.2)	16/72 (22.2)		9/35 (25.7)	8/53 (15.1)	
	NBCA + microparticles	2/16 (12.5)	13/72 (18.1)		4/35 (11.4)	11/53 (20.8)	
Post-PAE clinical evaluation							
Relative change in QOL at 3 months (%)		-	-	-	−37.2 ± 29.1	−66.6 ± 27.2	<0.0001 ***
Absolute change in QOL at 3 months		-	-	-	−1.8 ± 1.5	−3.1 ± 1.4	0.0001 ***
Relative change in IPSS at 3 months (%)		-	-	-	−34 ± 34.9	−61.9 ± 23.9	0.0002 ***
Absolute change in IPSS at 3 months		-	-	-	−7 ± 8	−11.5 ± 6.5	0.0045 **
Relative change in volume at 3 months		-	-	-	−15.8 ± 17.6	−18.1 ± 15.6	0.529
Absolute change in volume at 3 months (mL)		-	-	-	−14.2 ± 17.7	−20 ± 24.3	0.3181

NOTE—Data are numbers of patients with percentages in parentheses, except for numeric variables given as mean and standard deviation (SD). Other abbreviations: ADC: apparent diffusion coefficient, adBPH: adenomatous BPH, BPH: benign prostate hypertrophy, BMI: body mass index, IPSS: international prostate score symptom, NBCA: N-butyl cyanoacrylate glue, no.: number, PAE: prostate artery embolization, QOL: quality of life, ratio TZ/PZ: ratio between transitional zone and peripheral zone, WI: weighted imaging. Tests are unpaired Student *t*-test and Mann-Whitney Wilcoxon test, depending on the Shapiro–Wilk normality test. *: *p* < 0.05, **: *p* < 0.005, ***: *p* < 0.001.

As variations in QOL and IPSS were highly correlated (Spearman rho = 0.594, *p* < 0.0001), we selected the relative change in IPSS at 3 months for the multivariable analyses, in order to avoid collinearity issue, removing patients because of non-available data and because of lower *p*-value than absolute change in IPSS.

**Figure 5:**
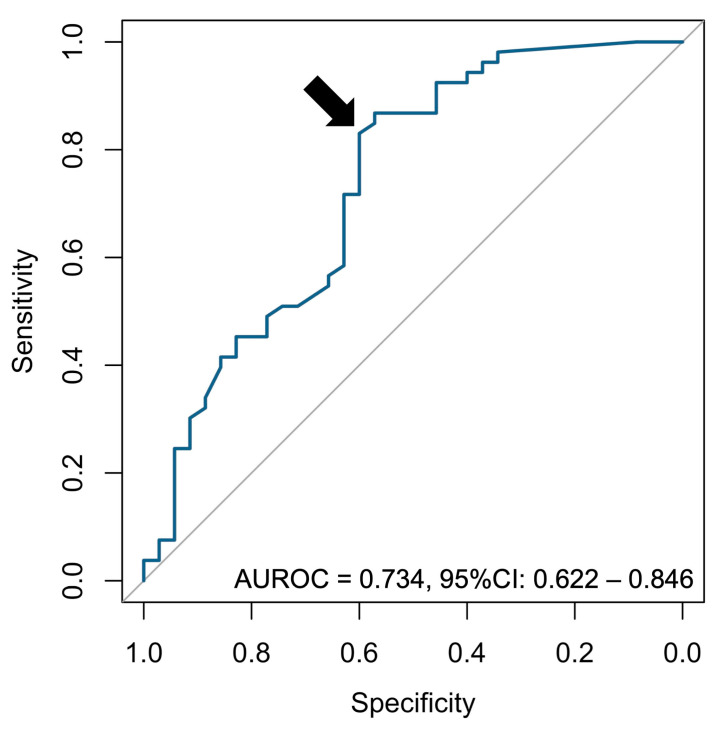
ROC curve analysis for the relative change in IPSS. The point corresponding to the Youden index (i.e., maximizing sensitivity + specificity −1) is indicated with a black arrow. Abbreviations: AUROC: area under the ROC curve, CI: confidence interval.

According to ROC analysis, the optimal cut-off for the relative changes in IPSS was −35% (AUROC = 0.734, 95%CI = 0.622–0.846, providing a sensitivity of 57% and a specificity of 87% to predict success at 2 years) (Figure 5).

Regarding the reproducibility of radiological features, the agreement was perfect for the presence of bladder lithiasis (α = 1), substantial for PPI (α = 0.6–0.8), moderate for adBPH, bladder diverticulum and Wasserman classification (α = 0.4–0.6), and fair for the signal on T2-WI (α = 0.2–0.4) (Table 3).

### 3.3. Radiomics Analyses

#### 3.3.1. Reproducible RFs

ADC maps of diagnostic quality were available in 82/88 (93.2%) patients. A total of 271 RFs were selected as reproducible, namely: 147/271 (54.2%) from T2-WI and 124/271 (45.8%) from ADC (Supplementary Table S2).

#### 3.3.2. Univariable Analyses (Figure 6A,B)

None of the RFs were associated with clinical success at 3 months or 2 years according to adjusted *p*-value < 0.05 and raw *p*-value < 0.05.

**Figure 6:**
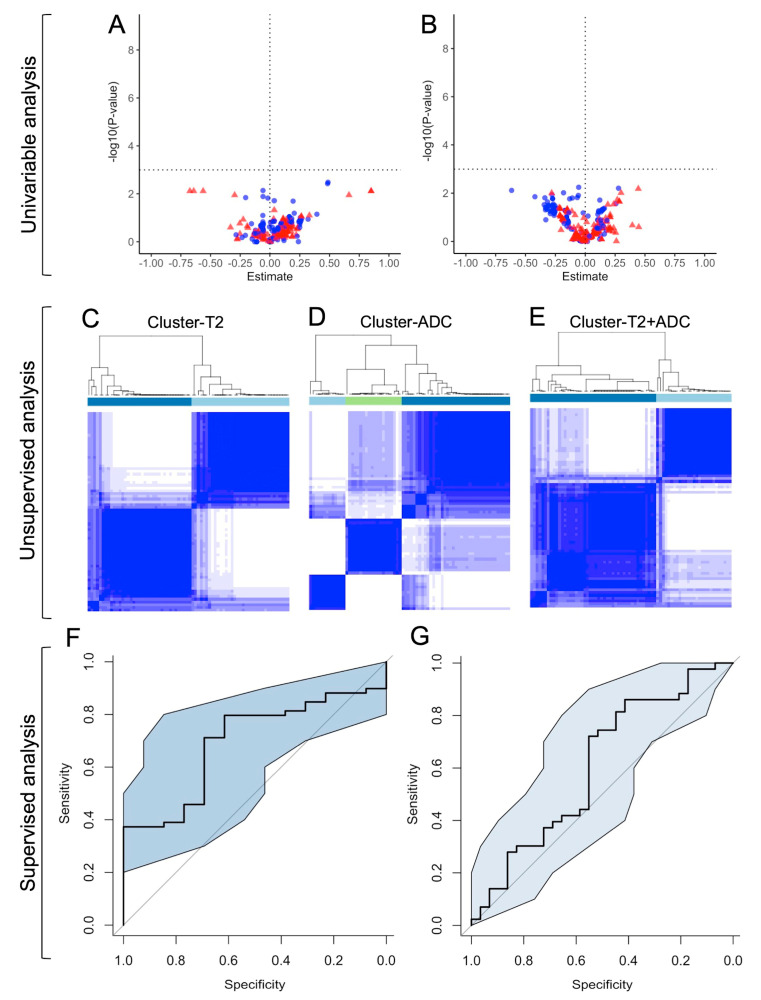
Summary of the radiomics analyses. The upper horizontal panel illustrates the univariable analysis. Volcano plots for the associations between the radiomics features (RFs) with clinical success at 3 months (M3) (**A**), and with clinical success at 2 years (Y2) (**B**). In the volcano plot, points located above the horizontal dotted line correspond to significant RFs. Points on the left of the vertical dotted line (negative Estimate, i.e., odds ratio [= exp(Estimate)]) < 1) are adverse predictors, and those on the right (positive Estimate, i.e., odds ratio > 1). Red points correspond to T2-based RFs, and blue points to ADC-based RFs. The middle horizontal panel corresponds to the unsupervised analysis and illustrates the consensual hierarchical clustering obtained on T2-based RFs (**C**), ADC-based RFs (**D**), and both ADC- and T2-based RFs (**E**). The lower horizontal panel shows the supervised analysis: cross-validated ROC curves with a 95% confidence interval (CI) for the best model at M3 (AUROC = 0.694, 95%CI = 0.552–0.836) (**F**), and at Y2 (AUROC = 0.607, 95%CI = 0.468–0.746) (**G**).

#### 3.3.3. Unsupervised Classifications

The unsupervised clustering provided three groups for cluster-ADC, two groups for cluster-T2, and two groups for cluster-T2+ADC (Figure 6C–E). The sole significant association was found between adBPH and cluster-T2 (adjusted *p*-value = 0.0291) (Supplementary Table S3, Figure 6C–E).

#### 3.3.4. Supervised Analyses

Regarding the clinical success at 3 months, the best predictive performance was obtained with the RFs from both T2-WI and ADC (AUROC = 0.694, 95%CI = 0.552–0.836, *p* = 0.0279), which did not significantly improve with PCA (*p* = 0.8695, Delong test) (Table 4, Figure 6F). The 23 selected variables per Elasticnet and their coefficients are given in Supplementary Table S4. After categorizing the corresponding RadScore-T2.ADCM3 per its median, its diagnostic accuracy was 0.569 (95%CI = 0.447–0.686).

At 2 years, the best predictive performance was also obtained with the RFs from both T2-WI and ADC (AUROC = 0.607, 95%CI = 0.468–0.746, *p* = 0.1273), which worsened with PCA (AUROC = 0.472, 95%CI = 0.373–0.607) (Table 4, Figure 6G). The RadScore-T2.ADCM3 was not associated with success at 2 years (*p* = 0.8195).

Models based on T2-WI alone and ADC alone did not provide significant results.

### 3.4. Multivariable Analyses

Since only the clinical success at 2 years provided more than one potential predictor, multivariable analyses were only conducted for this outcome (Table 5). The Wasserman type 1 was associated with clinical success at 2 years compared to the type 2 (OR = 6.59, 95%CI = 1.55–3.85, *p* = 0.0140, and OR = 6.47, 95%CI = 1.43–34.29, *p* = 0.0194 with confounding covariables). Similarly, the relative change in IPSS at 3 months was significantly associated with this outcome (OR = 0.97, 95%CI = 0.95–0.99, *p* = 0.0003, and OR = 0.97, 95%CI = 0.95–0.98, *p* = 0.0004 with confounding covariables).

**Figure 7:**
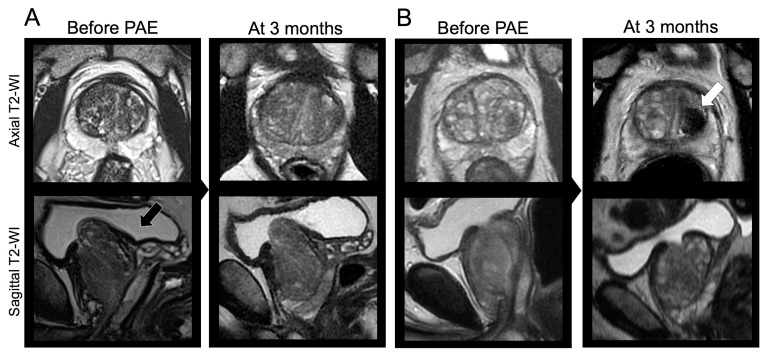
Clinical examples. (**A**) A 54-year-old male underwent a bilateral prostate artery embolization (PAE) with glue and microparticles for lower urinary tract symptoms (LUTS) due to benign prostate hypertrophy (BPH) with initial IPSS = 19 and initial QOL = 5. The prostate was classified as Wasserman type 2 (black arrow). The relative change in IPSS at 3 months was +21.9% (>−35%). The patient declared no relief from his symptoms at 3 months and 2 years following PAE. (**B**) A 64-year-old male underwent a bilateral PAE with microparticles for LUTS due to BPH with initial IPSS = 25 and initial QOL = 5, and a Wasserman type 1 prostate. At early revaluation, a prostate infarct was noticed (white arrow). The relative change in IPSS at 3 months was −92% (<−35%). The patient was satisfied with PAE and symptoms relief at 3 months, 2 years, and during his latest visit 38 months after PAE. Other abbreviation: WI: weighted imaging.

Figure 7 illustrates those findings through two opposite examples.

## 4. Discussion

We performed an exhaustive analysis of clinical, technical, radiological and radiomics features from the transitional zone of the prostate on pre-PAE MRI, in order to identify predictors of clinical success at 3 months and 2 years following technically successful PAE for LUTS due to BPH in 88 consecutive patients. The supervised radiomics scores were only weakly associated with clinical success at 3 months without predictive value at 2 years. However, the early changes in IPSS and QOL, and the Wasserman classification were associated with mid-term clinical success.

First, our results confirm the safety and efficacy of PAE for LUTS due to BPH. The average initial IPSS and early and latest absolute changes in IPSS were 18.7, −9.8, and, −7.3, respectively, versus an initial IPSS of 22.4 and a decrease at 6 months of −10.5 in the study by Bilhim et al. [8], versus an initial IPSS of 20.3 and a reduction at 4 months of −9.5 according to Frandon et al. [11]. The rates of adverse events were similar with 15.9% of patients reporting such issues, including 3% of major complications versus 14.6% of minor complications and 1.3% of major complications in the study by Frandon et al. [11].

The rates of clinical success were 81.8% at 3 months and 60.2% at 2 years, which is below the rates reported in prior studies. Indeed, Pisco et al. found clinical success in 90% of patients at 3 months, and 81.9% of patients at 2 years in a cohort [4]. On the other hand, we observed a recurrence rate of 25% at 2 years, which is in line with the 21% of patients who required prostate surgery within 2 years following PAE [6]. Interestingly, we observed 3.4% of patients who were not relieved at 3 months but later, suggesting the possibility of delayed response following PAE.

Regarding the short-term clinical, no pre-PAE clinical or radiological variable was associated with this outcome. However, at 2 years, the multivariable analysis highlighted the better outcome in patients with prostate classified as Wasserman type 1 compared to type 2 (multivariable OR = 5.82, when accounting for age and embolization material and after categorizing changes in IPSS). Wasserman type 1 corresponds to a simple hypertrophy of the transitional zone whereas type 2 to a retro-urethral or pedunculated hypertrophy alone (i.e., an isolated hypertrophic median lobe) [10,18]. This association was not significant in the study by Boschheidgen et al., but their population was smaller (N = 66 versus N = 88 in our study) [10]. Surprisingly, we did not observe a significant association between success and PPI. Yet, as explained by Yu et al. [14], PPI gathers all types of median lobe and all types of PPI coming from other prostatic areas. We report 15.9% of patients with median lobe and 39% of patients with PPI grade 3, which illustrates the incomplete overlap between those two characteristics. The poor prognosis of the median lobe would be related to a ‘ball valve’ effect [14,26]. In this theory, PEA would lead to softer and more mobile prostate tissue due to ischemic alterations, which would be responsible for an obstruction of the proximal urethra in the case of an embolized median lobe [14,26].

We purposely selected patients with technical success to focus on the pre-treatment radiological and radiomics characteristics. This point may explain why some variables were not associated with clinical successes such as embolization material, prostate volume, or cardiovascular comorbidities [11].

The early changes in IPSS at 3 months were strongly associated with clinical success at 2 years, in line with prior studies [7,11]. We identified a cut-off of −35% decrease in IPSS, which could be more clinically meaningful in practice compared to continuous value. The multivariable OR for mid-term clinical success was 8.82 for patients with a decrease in IPSS at 3 months ≤ −35% compared to others (accounting for Wasserman classification, embolization material, and age). Unfortunately, the relative change in IPSS at 3 months is unhelpful to select patients for PAE before the procedure.

The adBPH feature was reported in 58% of patients from our study but did not correlate with successes. The links between adBPH and good outcomes after PAE remain controversial and our results agree with those of Abt et al. [12].

The originality of our work corresponds to the radiomics analysis. We designed a radiomics workflow following the guidelines to limit the risk of false discoveries due to multidimensional data [15,16,27,28]. Overall, its conclusions were disappointing. No association was found with clinical success at 2 years and the clinical relevance of the best model (combining RFs from T2-WI and ADC) for success at 3 months was limited, with an AUROC < 0.7 and a diagnostic accuracy < 0.6 after dichotomization. However, we noticed a significant association between the T2-based radiomics cluster and adBPH. This is not the first time that radiomics fails and failures of radiomics should also be published to prevent other groups from re-performing the same negative study [27]. To our opinion, it may be due to (i) heterogeneous MRI datasets (although we performed post-processing to reduce this heterogeneity), (ii) shape RFs that are unable to discriminate the median lobe, and (iii) lack of associations between adenoma texture and patients’ outcomes.

Our study has limitations. First, it was a retrospective, single-center study on a rather small and selected population. However, we included all consecutive real-life patients addressed to a center of expertise. Moreover, the descriptive characteristics of the population were comparable with those of prior studies. A consequence of this retrospective design was the lack of standardization of the delays and contents of the follow-up visits. Hence, we did not know the IPSS value at exactly 2 years, which should be comprised between the IPSS at 3 months and the IPSS at the end of the study (i.e., more than 2 years after the PAE procedure) that were both available. Second, the follow-up was not standardized after the medical visit at 3 months and remains limited to 6 years after PAE at best. Third, the lack of a standardized definition for clinical success is a real issue for comparing studies. We used a composite assessment with the overall patients’ satisfaction without the need for other treatments or interventions being the key to this evaluation, but an international and consensual definition would be desirable. Fourth, we did not validate our radiomics models on an independent test set, but the disappointing results in cross-validation did not deserve further investigation.

## 5. Conclusions

To conclude, this study highlights the feasibility of radiomics analyses in the setting of an interventional radiology study for benign pathology. However, radiomics failed to predict clinical successes after PAE for LUTS due to BPH. Radiomics features rather seemed to correlate with adBPH, which failed to predict patients’ outcomes. Nonetheless, our study strengthens the predictive value of the early relative change in IPSS and the potential of the Wasserman classification to identify good candidates for PAE, emphasizing the need for reproducible quantitative indices reflecting the Wasserman classification.

## Figures and Tables

**Table 1:** Characteristics of the study population.

Characteristics		Patients	No. of Missing Data
Age (years)		65 [62–70] (54–84)	0
Age (categorized)			
	≥75 years	11/88 (12.5)	0
	65–74 years	37/88 (42)	
	<65 years	40/88 (45.5)	
Medical treatment for BPH		63/86 (73.3)	2
BMI groups			
	Obese (≥30 kg·m^−2^)	11/83 (13.3)	5
	Surpoids (25–29 kg·m^−2^)	36/83 (43.4)	
	Normal (<25 kg·m^−2^)	36/83 (43.4)	
Cardiovascular comorbidities		35/85 (41.2)	3
Need for 2nd PAE procedure		4/88 (4.5)	0
Embolization material			
	NBCA	56/88 (63.6)	0
	Microparticle	17/88 (19.3)	
	NBCA + microparticle	15/88 (17)	
Initial prostate volume (mL)		80 [60–122] (38–200)	0
Initial QOL		5 [4–6] (1–6)	1
Initial IPSS		18.7 ± 6.3 (7–32)	0

NOTE—Data are numbers of patients with percentages in parentheses, except for numeric variables given as median, interquartile range (in bracket), and range (in parentheses) or mean, standard deviation, and range, depending on Shapiro-Wilk normality test. Abbreviations: BPH: benign prostate hypertrophy, BMI: body mass index, IPSS: international prostate score symptom, NBCA: N-butyl cyanoacrylate glue, no.: number, PAE: prostate artery embolization, QOL: quality of life.

**Table 3:** Reproducibility analysis of the radiological features over the three radiologists.

Radiological Features	Krippendorf’s Alpha
Prostate protrusion index ^§^	0.727 (0.639–0.840)
Adenomatous dominant benign prostate hypertrophy	0.587 (0.459–0.727)
Signal intensity on T2-weighted imaging ^§^	0.352 (0.228–0.487)
Wasserman classification	0.492 (0.370–0.626)
Bladder diverticulum	1.000 (1.000–1.000)
Bladder lithiasis	0.577 (0.423–0.704)

NOTE—Krippendorf’s alphas are given with 95% confidence intervals. §: ordinal variable. Of note, alpha values are similar to Fleiss Kappa for nominal variables.

**Table 4:** Results of the supervised Elasticnet logistic regression models.

Endpoint	Radiomics Features	PCA	α	λ	AUROC (95%CI)	*p*-Value
M3	T2-WI	no	0.025	5	0.475 (0.331–0.620)	0.7327
		yes	0.025	5	0.475 (0.331–0.620)	0.7327
	ADC	no	0	0	0.588 (0.409–0.767)	0.3267
		yes	0.025	0	0.606 (0.428–0.784)	0.2357
	T2-WI + ADC	no	0.55	0	0.694 (0.552–0.836)	0.0279 *
		yes	0.15	0	0.704 (0.579–0.829)	0.0224 *
Y2	T2-WI	no	0.025	1.6	0.488 (0.361–0.616)	0.8579
		yes	0.025	5	0.489 (0.370–0.608)	0.8545
	ADC	no	0	3.4	0.509 (0.362–0.657)	0.9002
		yes	0.05	5	0.472 (0.337–0.607)	0.6860
	T2-WI + ADC	no	0.075	0	0.607 (0.468–0.747)	0.1273
		yes	0.05	5	0.472 (0.373–0.607)	0.6860

NOTE—Abbreviations: ADC: apparent diffusion coefficient, AUROC: area under the ROC curve, CI: confidence interval, M3: clinical success at 3 months, PCA: with principal component analysis, WI: weighted imaging, Y2: clinical success at 2 years. *: *p* < 0.05. Hyperparameters: α: controls the elasticnet penalty and bridges the gap between LASSO regression (α = 1, the default) and ridge regression (α = 0); λ: controls the overall strength of the penalty.

**Table 5:** Multivariable analyses for clinical success at 2 years after prostate artery embolization for benign prostate hypertrophy.

Characteristics		Multivariable Analysis		Multivariable Analysis with Covariates	
		OR (95%CI)	*p*-Value	OR (95%CI)	*p*-Value
With raw numeric variables					
Wasserman classification (ref: type 2)					
	Type 1	6.59 (1.55–32.85)	0.0140 *	6.47 (1.43–34.29)	0.0194 *
	Type 3	4.36 (1.08–20.69)	0.0466 *	3.66 (0.86–17.77)	0.0869
Relative change in IPSS (continuous)		0.97 (0.95–0.99)	0.0003 ***	0.97 (0.95–0.98)	0.0004 ***
Age groups (ref: ≥75 y)					
	<65 y	-	-	0.71 (0.14–3.43)	0.6621
	65 y–74 y	-	-	1.43 (0.26–7.20)	0.6679
Embolization material (ref: NBCA)					
	Microparticles alone	-	-	0.40 (0.10–1.54)	0.1839
	NBCA + Microparticle	-	-	0.81 (0.19–3.85)	0.7824
After categorization					
Wasserman classification (ref: type 2)					
	Type 1	6.14 (1.40–31)	0.0196 *	5.82 (1.25–31.29)	0.0296 *
	Type 3	4.47 (1.09–21.26)	0.0446 *	3.75 (0.87–18.60)	0.0850
Relative change in IPSS (categorized, ref: ≤−35%)		8.68 (3.06–27.31)	<0.0001 ***	8.82 (2.92–29.97)	0.0002 ***
Age groups (ref: ≥75 y)					
	<65 y	-	-	0.61 (0.11–2.94)	0.5416
	65 y–74 y	-	-	1.07 (0.19–5.38)	0.9375
Embolization material (ref: NBCA)					
	Microparticles alone	-	-	0.47 (0.12–1.81)	0.2727
	NBCA + Microparticle	-	-	0.88 (0.20–4.26)	0.8716

NOTE—*: *p* < 0.05, ***: *p* < 0.001. Abbreviations: CI: confidence interval, IPSS: international prostate score symptom, NBCA: N-butyl cyanoacrylate glue, OR: odds ratio.

## Data Availability

The dataset, LIFEx scripts, and R codes used to generate the results can be available from the corresponding authors upon reasonable request.

## Supplementary Materials

The following supporting information can be downloaded at: https://www.mdpi.com/article/10.3390/jpm14010067/s1: Table S1: Changes in prostate volume, IPSS, and QOL in the entire cohort. Table S2: Reproducibility analysis of the radiomics features (i.e., intraclass correlation coefficient [ICC] and 95% confidence interval [CI]) Table S3: Understanding unsupervised radiomics clusters Table S4: Radiomics features selected by Elasticnet.
