# Supplementary material for: Clinical, Technical, and MRI Features Associated with Patients’ Outcome at 3 Months and 2 Years following Prostate Artery Embolization: Is There an Added Value of Radiomics?
Source: J Pers Med. 2024 Jan 4;14(1):67. doi: 10.3390/jpm14010067 (PMC10817287; doi:10.3390/jpm14010067)
Supplement: Supplementary file 1 [file jpm-14-00067-s001.zip › jpm-2701652-supplementary.pdf]

**SUPPLEMENTARY MATERIAL**

**Supplementary Table S1.** Changes in prostate volume, IPSS and QOL in the entire cohort.

| Enpoint | Mean ± SD | Median [Q1-Q3] (min-max) | No. Of missing values |
|---|---|---|---|
| **QOL** | | | |
| Initial QOL before PAE | 4.7 ± 1.1 | 5 [4 - 6] (1 - 6) | 1 |
| QOL at M3 | 2.1 ± 1.6 | 2 [1 - 3] (0 - 6) | 1 |
| QOL at last FU | 2.5 ± 1.8 | 3 [1 -4] (1 - 4) | 2 |
| Absolute change in QOL from baseline to M3 | -2.6 ± 1.6 | -3 [-4 - -1] (-6 - 0) | 1 |
| Relative change in QOL from baseline to M3 (%) | -54.8 ± 31.4 | -60 [-77.5 - -25] (-100 - 0) | 1 |
| Absolute change in QOL from baseline to last FU | -2.2 ± 2 | -2 [-4 - -0.2] (-6 - 4) | 2 |
| Relative change in QOL from baseline to last FU (%) | -43.5 ± 47.2 | -50 [-80 - -4.2] (-100 - 200) | 2 |
| **IPSS** | | | |
| Initial IPSS before PAE | 18.7 ± 6.3 | 19 [13 - 23] (8 - 32) | 0 |
| IPSS at M3 | 8.9 ± 6.8 | 7 [4 - 12] (1 - 33) | 0 |
| IPSS at last FU | 11.4 ± 7.6 | 10 [5 - 15] (0 - 28) | 1 |
| Absolute change in IPSS from baseline to M3 | -9.8 ± 7.4 | -10 [-15 - -4] (-31 - 5) | 0 |
| Relative change in IPSS from baseline to M3 (%) | -50.8 ± 31.7 | -59.1 [-75.3 - -30.2] (-96.9 – 21.7) | 0 |
| Absolute change in IPSS from baseline to last FU | -7.3 ± 7.8 | -7 [-13 - -2.5] (-24 - 17) | 1 |
| Relative change in IPSS from baseline to last FU (%) | -36.6 ± 45.9 | -43.8 [-66.7 - -16.1] (-100 - 212.5) | 1 |
| **Prostate volume** | | | |
| Initial volume before PAE | 92.1 ± 40.7 | 80 [60 - 122.5] (38 - 200) | 0 |
| Volume at M3 | 74.3 ± 31.8 | 65 [50 - 99] (25 - 162) | 0 |
| Absolute change in volume from baseline to M3 | -17.7 ± 22 | -11 [-25 - -3] (-120 - 15) | 0 |
| Relative change in volume from baseline to M3 (%) | -17.2 ± 16.4 | -17.4 [-25.5 - -5.6] (-61.5 - 25.4) | 0 |

NOTE.- Abbreviations: FU: follow-up (at least 2 years), IPSS: international prostate score symptom, no.: number, M3: at 3 months after prostate artery embolization, PAE: prostate artery embolization, QOL: quality of life

**Supplementary Table S2.** Reproducibility analysis of the radiomics features (i.e., intraclass correlation coefficient [ICC] and 95% confidence interval [CI])

| Radiomics features | ICC (95%CI) | *P*-value |
|---|---|---|
| ADC_GLRLM_LowGreyLevelRunEmphasis | 1 (1 - 1) | <0.0001*** |
| ADC_GLSZM_LowGrayLevelZoneEmphasis | 1 (1 - 1) | <0.0001*** |
| ADC_GLCM2_AngularSecondMoment | 1 (1 - 1) | <0.0001*** |
| ADC_GLCM_AngularSecondMoment | 1 (1 - 1) | <0.0001*** |
| ADC_GLCM4_AngularSecondMoment | 1 (1 - 1) | <0.0001*** |
| ADC_GLCM2_JointMaximum | 1 (1 - 1) | <0.0001*** |
| ADC_GLCM_JointMaximum | 1 (1 - 1) | <0.0001*** |
| ADC_GLCM4_JointMaximum | 1 (1 - 1) | <0.0001*** |
| ADC_GLSZM_NormalisedGreyLevelNonUniformity | 1 (1 - 1) | <0.0001*** |
| ADC_INTENSITY-HISTOGRAM_UniformityIBSI | 1 (1 - 1) | <0.0001*** |
| ADC_GLCM2_InverseDifferenceMoment | 1 (0.999 - 1) | <0.0001*** |
| ADC_INTENSITY-BASED_75thPercentile | 1 (0.999 - 1) | <0.0001*** |
| ADC_GLCM_InverseDifferenceMoment | 1 (0.999 - 1) | <0.0001*** |
| ADC_INTENSITY-HISTOGRAM_INTENSITYHistogram75thPercentile | 0.999 (0.999 - 1) | <0.0001*** |
| ADC_GLCM2_InverseDifference | 0.999 (0.999 - 1) | <0.0001*** |
| ADC_GLRLM_RunPercentage | 0.999 (0.999 - 1) | <0.0001*** |
| ADC_GLCM2_JointAverage | 0.999 (0.999 - 1) | <0.0001*** |
| ADC_GLCM2_SumAverage | 0.999 (0.999 - 1) | <0.0001*** |
| ADC_GLCM_InverseDifference | 0.999 (0.999 - 1) | <0.0001*** |
| ADC_GLCM_JointAverage | 0.999 (0.999 - 0.999) | <0.0001*** |
| ADC_GLCM_SumAverage | 0.999 (0.999 - 0.999) | <0.0001*** |
| ADC_INTENSITY-BASED_RootMeanSquare | 0.999 (0.999 - 0.999) | <0.0001*** |
| ADC_GLCM4_JointAverage | 0.999 (0.999 - 0.999) | <0.0001*** |
| ADC_GLCM4_SumAverage | 0.999 (0.999 - 0.999) | <0.0001*** |
| ADC_INTENSITY-BASED_Median | 0.999 (0.999 - 0.999) | <0.0001*** |
| ADC_INTENSITY-BASED_50thPercentile | 0.999 (0.999 - 0.999) | <0.0001*** |
| ADC_INTENSITY-BASED_Mean | 0.999 (0.999 - 0.999) | <0.0001*** |
| ADC_INTENSITY-BASED_AreaUnderCurve | 0.999 (0.999 - 0.999) | <0.0001*** |
| ADC_INTENSITY-HISTOGRAM_AreaUnderCurveCsh | 0.999 (0.999 - 0.999) | <0.0001*** |
| ADC_INTENSITY-HISTOGRAM_INTENSITYHistogramMean | 0.999 (0.999 - 0.999) | <0.0001*** |
| ADC_INTENSITY-HISTOGRAM_INTENSITYHistogramMedian | 0.999 (0.998 - 0.999) | <0.0001*** |
| ADC_INTENSITY-HISTOGRAM_INTENSITYHistogram50thPercentile | 0.999 (0.998 - 0.999) | <0.0001*** |
| ADC_INTENSITY-BASED_90thPercentile | 0.999 (0.998 - 0.999) | <0.0001*** |
| ADC_INTENSITY-HISTOGRAM_INTENSITYHistogram90thPercentile | 0.999 (0.998 - 0.999) | <0.0001*** |
| ADC_GLCM2_Autocorrelation | 0.999 (0.998 - 0.999) | <0.0001*** |
| ADC_GLCM_Autocorrelation | 0.999 (0.998 - 0.999) | <0.0001*** |
| ADC_GLRLM_ShortRunsEmphasis | 0.998 (0.997 - 0.999) | <0.0001*** |
| ADC_GLRLM_LongRunHighGreyLevelEmphasis | 0.998 (0.997 - 0.999) | <0.0001*** |
| ADC_GLRLM_HighGreyLevelRunEmphasis | 0.998 (0.997 - 0.999) | <0.0001*** |
| ADC_GLRLM_ShortRunHighGreyLevelEmphasis | 0.998 (0.997 - 0.999) | <0.0001*** |
| ADC_INTENSITY-BASED_25thPercentile | 0.998 (0.997 - 0.999) | <0.0001*** |
| ADC_GLCM4_Autocorrelation | 0.998 (0.996 - 0.998) | <0.0001*** |
| ADC_INTENSITY-HISTOGRAM_INTENSITYHistogram25thPercentile | 0.998 (0.996 - 0.998) | <0.0001*** |
| ADC_GLCM_DifferenceEntropy | 0.997 (0.996 - 0.998) | <0.0001*** |
| ADC_GLCM_JointEntropyLog2 | 0.997 (0.996 - 0.998) | <0.0001*** |
| ADC_GLCM_JointEntropyLog10 | 0.997 (0.996 - 0.998) | <0.0001*** |
| ADC_GLCM2_JointEntropyLog2 | 0.997 (0.996 - 0.998) | <0.0001*** |
| ADC_GLCM2_DifferenceEntropy | 0.997 (0.996 - 0.998) | <0.0001*** |
| ADC_GLCM2_JointEntropyLog10 | 0.997 (0.996 - 0.998) | <0.0001*** |
| ADC_GLSZM_ZoneSizeEntropy | 0.997 (0.995 - 0.998) | <0.0001*** |
| ADC_GLSZM_HighGrayLevelZoneEmphasis | 0.997 (0.995 - 0.998) | <0.0001*** |
| ADC_GLSZM_SmallZoneEmphasis | 0.996 (0.994 - 0.998) | <0.0001*** |
| T2_GLSZM_LargeZoneLowGreyLevelEmphasis | 0.996 (0.994 - 0.997) | <0.0001*** |
| ADC_INTENSITY-HISTOGRAM_INTENSITYHistogramEntropyLog2 | 0.996 (0.994 - 0.997) | <0.0001*** |
| ADC_INTENSITY-HISTOGRAM_INTENSITYHistogramEntropyLog10 | 0.996 (0.994 - 0.997) | <0.0001*** |
| ADC_GLSZM_ZonePercentage | 0.994 (0.992 - 0.996) | <0.0001*** |
| T2_GLSZM_LargeZoneHighGreyLevelEmphasis | 0.994 (0.991 - 0.996) | <0.0001*** |
| ADC_GLSZM_SmallZoneHighGreyLevelEmphasis | 0.994 (0.991 - 0.996) | <0.0001*** |
| ADC_INTENSITY-BASED_10thPercentile | 0.994 (0.991 - 0.996) | <0.0001*** |
| T2_GLCM2_Autocorrelation | 0.993 (0.99 - 0.996) | <0.0001*** |
| ADC_INTENSITY-HISTOGRAM_INTENSITYHistogram10thPercentile | 0.993 (0.99 - 0.995) | <0.0001*** |
| T2_GLCM4_Autocorrelation | 0.993 (0.989 - 0.995) | <0.0001*** |
| ADC_INTENSITY-HISTOGRAM_INTENSITYHistogramModeI | 0.993 (0.989 - 0.995) | <0.0001*** |
| T2_GLSZM_ZoneSizeVariance | 0.993 (0.989 - 0.995) | <0.0001*** |

| | | |
|---|---|---|
| T2_GLCM_Autocorrelation | 0.992 (0.989 - 0.995) | <0.0001*** |
| T2_GLSZM_LargeZoneEmphasis | 0.992 (0.989 - 0.995) | <0.0001*** |
| ADC_GLSZM_NormalisedZoneSizeNonUniformity | 0.992 (0.989 - 0.995) | <0.0001*** |
| T2_GLCM2_JointAverage | 0.992 (0.989 - 0.995) | <0.0001*** |
| T2_GLCM2_SumAverage | 0.992 (0.989 - 0.995) | <0.0001*** |
| T2_GLCM4_JointAverage | 0.992 (0.988 - 0.995) | <0.0001*** |
| T2_GLCM4_SumAverage | 0.992 (0.988 - 0.995) | <0.0001*** |
| ADC_GLCM4_DifferenceEntropy | 0.992 (0.987 - 0.994) | <0.0001*** |
| ADC_GLCM4_JointEntropyLog2 | 0.992 (0.987 - 0.994) | <0.0001*** |
| ADC_GLCM4_JointEntropyLog10 | 0.992 (0.987 - 0.994) | <0.0001*** |
| T2_INTENSITY-BASED_Median | 0.991 (0.987 - 0.994) | <0.0001*** |
| T2_INTENSITY-BASED_50thPercentile | 0.991 (0.987 - 0.994) | <0.0001*** |
| T2_INTENSITY-BASED_75thPercentile | 0.991 (0.987 - 0.994) | <0.0001*** |
| T2_GLCM_JointAverage | 0.991 (0.987 - 0.994) | <0.0001*** |
| T2_GLCM_SumAverage | 0.991 (0.987 - 0.994) | <0.0001*** |
| T2_GLRLM_LongRunHighGreyLevelEmphasis | 0.991 (0.986 - 0.994) | <0.0001*** |
| T2_INTENSITY-BASED_90thPercentile | 0.991 (0.986 - 0.994) | <0.0001*** |
| T2_INTENSITY-HISTOGRAM_INTENSITYHistogramMedian | 0.991 (0.986 - 0.994) | <0.0001*** |
| T2_INTENSITY-HISTOGRAM_INTENSITYHistogram50thPercentile | 0.991 (0.986 - 0.994) | <0.0001*** |
| T2_INTENSITY-HISTOGRAM_INTENSITYHistogram75thPercentile | 0.991 (0.986 - 0.994) | <0.0001*** |
| T2_INTENSITY-HISTOGRAM_INTENSITYHistogram90thPercentile | 0.99 (0.985 - 0.993) | <0.0001*** |
| T2_INTENSITY-BASED_RootMeanSquare | 0.99 (0.985 - 0.993) | <0.0001*** |
| T2_GLRLM_HighGreyLevelRunEmphasis | 0.99 (0.985 - 0.993) | <0.0001*** |
| T2_GLRLM_ShortRunHighGreyLevelEmphasis | 0.989 (0.984 - 0.993) | <0.0001*** |
| T2_GLRLM_LongRunsEmphasis | 0.988 (0.982 - 0.992) | <0.0001*** |
| T2_GLRLM_RunPercentage | 0.988 (0.982 - 0.992) | <0.0001*** |
| T2_INTENSITY-BASED_AreaUnderCurve | 0.988 (0.981 - 0.992) | <0.0001*** |
| T2_INTENSITY-BASED_Mean | 0.988 (0.981 - 0.992) | <0.0001*** |
| T2_GLSZM_ZonePercentage | 0.988 (0.981 - 0.992) | <0.0001*** |
| T2_INTENSITY-HISTOGRAM_AreaUnderCurveCsh | 0.988 (0.981 - 0.992) | <0.0001*** |
| T2_INTENSITY-HISTOGRAM_INTENSITYHistogramMean | 0.988 (0.981 - 0.992) | <0.0001*** |
| T2_GLRLM_ShortRunsEmphasis | 0.987 (0.981 - 0.991) | <0.0001*** |
| T2_GLCM_InverseDifference | 0.987 (0.98 - 0.991) | <0.0001*** |
| T2_GLCM_InverseDifferenceMoment | 0.987 (0.98 - 0.991) | <0.0001*** |
| T2_GLSZM_HighGrayLevelZoneEmphasis | 0.987 (0.98 - 0.991) | <0.0001*** |
| T2_GLCM2_Contrast | 0.987 (0.98 - 0.991) | <0.0001*** |
| T2_GLCM_DifferenceAverage | 0.986 (0.979 - 0.991) | <0.0001*** |
| T2_GLCM_Dissimilarity | 0.986 (0.979 - 0.991) | <0.0001*** |
| T2_GLCM4_Contrast | 0.986 (0.979 - 0.99) | <0.0001*** |
| T2_INTENSITY-BASED_25thPercentile | 0.986 (0.978 - 0.99) | <0.0001*** |
| T2_GLCM2_DifferenceAverage | 0.985 (0.978 - 0.99) | <0.0001*** |
| T2_GLCM2_Dissimilarity | 0.985 (0.978 - 0.99) | <0.0001*** |
| T2_GLSZM_SmallZoneHighGreyLevelEmphasis | 0.985 (0.978 - 0.99) | <0.0001*** |
| T2_GLCM_Contrast | 0.985 (0.977 - 0.99) | <0.0001*** |
| T2_INTENSITY-HISTOGRAM_INTENSITYHistogramModeI | 0.984 (0.977 - 0.99) | <0.0001*** |
| T2_GLCM2_JointVariance | 0.984 (0.976 - 0.99) | <0.0001*** |
| ADC_GLCM_DifferenceAverage | 0.984 (0.976 - 0.989) | <0.0001*** |
| ADC_GLCM_Dissimilarity | 0.984 (0.976 - 0.989) | <0.0001*** |
| T2_GLCM2_DifferenceVariance | 0.984 (0.976 - 0.989) | <0.0001*** |
| T2_GLCM4_DifferenceVariance | 0.984 (0.976 - 0.989) | <0.0001*** |
| T2_GLCM_JointVariance | 0.984 (0.976 - 0.989) | <0.0001*** |
| T2_INTENSITY-HISTOGRAM_INTENSITYHistogram25thPercentile | 0.983 (0.975 - 0.989) | <0.0001*** |
| T2_GLCM2_InverseDifference | 0.983 (0.975 - 0.989) | <0.0001*** |
| T2_GLCM4_JointVariance | 0.983 (0.974 - 0.989) | <0.0001*** |
| T2_GLCM2_InverseDifferenceMoment | 0.983 (0.974 - 0.989) | <0.0001*** |
| T2_INTENSITY-HISTOGRAM_MaximumHistogramGradientGreyLevel | 0.982 (0.974 - 0.988) | <0.0001*** |
| T2_GLCM_SumVariance | 0.982 (0.974 - 0.988) | <0.0001*** |
| T2_GLCM_ClusterTendency | 0.982 (0.974 - 0.988) | <0.0001*** |
| ADC_GLCM2_Correlation | 0.982 (0.972 - 0.988) | <0.0001*** |
| T2_GLCM4_DifferenceAverage | 0.981 (0.971 - 0.987) | <0.0001*** |
| T2_GLCM4_Dissimilarity | 0.981 (0.971 - 0.987) | <0.0001*** |
| ADC_INTENSITY-HISTOGRAM_MaximumHistogramGradientGreyLevel | 0.981 (0.971 - 0.987) | <0.0001*** |
| T2_GLCM2_SumVariance | 0.98 (0.971 - 0.987) | <0.0001*** |
| T2_GLCM2_ClusterTendency | 0.98 (0.971 - 0.987) | <0.0001*** |
| ADC_GLCM2_DifferenceAverage | 0.979 (0.969 - 0.986) | <0.0001*** |
| ADC_GLCM2_Dissimilarity | 0.979 (0.969 - 0.986) | <0.0001*** |
| T2_GLCM_DifferenceVariance | 0.978 (0.967 - 0.985) | <0.0001*** |
| T2_GLCM4_SumVariance | 0.978 (0.967 - 0.985) | <0.0001*** |
| T2_GLCM4_ClusterTendency | 0.978 (0.967 - 0.985) | <0.0001*** |
| ADC_GLCM4_InverseDifferenceMoment | 0.978 (0.966 - 0.985) | <0.0001*** |

| | | |
|---|---|---|
| ADC_GLRLM_LongRunLowGreyLevelEmphasis | 0.978 (0.966 - 0.985) | <0.0001*** |
| ADC_INTENSITY-HISTOGRAM_INTENSITYHistogramRobustMeanAbsoluteDeviation | 0.977 (0.965 - 0.985) | <0.0001*** |
| ADC_GLRLM_LongRunsEmphasis | 0.977 (0.965 - 0.985) | <0.0001*** |
| ADC_INTENSITY-BASED_InterquartileRange | 0.976 (0.964 - 0.984) | <0.0001*** |
| ADC_INTENSITY-BASED_RobustMeanAbsoluteDeviation | 0.976 (0.963 - 0.984) | <0.0001*** |
| T2_INTENSITY-HISTOGRAM_INTENSITYHistogramRobustMeanAbsoluteDeviation | 0.974 (0.961 - 0.982) | <0.0001*** |
| T2_GLCM4_InverseDifference | 0.973 (0.96 - 0.982) | <0.0001*** |
| ADC_INTENSITY-HISTOGRAM_INTENSITYHistogramInterquartileRange | 0.973 (0.959 - 0.982) | <0.0001*** |
| T2_INTENSITY-BASED_InterquartileRange | 0.973 (0.96 - 0.982) | <0.0001*** |
| T2_INTENSITY-BASED_RobustMeanAbsoluteDeviation | 0.973 (0.959 - 0.982) | <0.0001*** |
| T2_INTENSITY-HISTOGRAM_INTENSITYHistogramVariance | 0.972 (0.959 - 0.981) | <0.0001*** |
| T2_INTENSITY-BASED_Variance | 0.972 (0.959 - 0.981) | <0.0001*** |
| T2_GLCM4_InverseDifferenceMoment | 0.972 (0.958 - 0.981) | <0.0001*** |
| ADC_GLCM_Correlation | 0.97 (0.955 - 0.98) | <0.0001*** |
| T2_GLCM_ClusterShade | 0.97 (0.955 - 0.98) | <0.0001*** |
| ADC_GLCM4_InverseDifference | 0.97 (0.954 - 0.98) | <0.0001*** |
| ADC_INTENSITY-HISTOGRAM_INTENSITYHistogramQuartileCoefficientOfDispersion | 0.969 (0.953 - 0.979) | <0.0001*** |
| T2_INTENSITY-BASED_MeanAbsoluteDeviation | 0.967 (0.951 - 0.978) | <0.0001*** |
| T2_INTENSITY-HISTOGRAM_INTENSITYHistogramMeanAbsoluteDeviation | 0.967 (0.951 - 0.978) | <0.0001*** |
| T2_GLCM_JointMaximum | 0.967 (0.951 - 0.978) | <0.0001*** |
| T2_GLCM2_ClusterShade | 0.966 (0.949 - 0.977) | <0.0001*** |
| T2_GLCM_ClusterProminence | 0.966 (0.949 - 0.977) | <0.0001*** |
| T2_INTENSITY-HISTOGRAM_INTENSITYHistogram10thPercentile | 0.965 (0.948 - 0.977) | <0.0001*** |
| T2_GLSZM_NormalisedZoneSizeNonUniformity | 0.965 (0.948 - 0.977) | <0.0001*** |
| T2_GLCM4_JointMaximum | 0.964 (0.947 - 0.976) | <0.0001*** |
| ADC_INTENSITY-HISTOGRAM_RootMeanSquare | 0.964 (0.946 - 0.977) | <0.0001*** |
| T2_INTENSITY-BASED_MedianAbsoluteDeviation | 0.964 (0.947 - 0.976) | <0.0001*** |
| T2_INTENSITY-HISTOGRAM_INTENSITYHistogramMedianAbsoluteDeviation | 0.964 (0.947 - 0.976) | <0.0001*** |
| T2_INTENSITY-BASED_10thPercentile | 0.964 (0.946 - 0.976) | <0.0001*** |
| T2_GLCM2_ClusterProminence | 0.964 (0.946 - 0.976) | <0.0001*** |
| T2_GLCM4_ClusterShade | 0.963 (0.946 - 0.976) | <0.0001*** |
| T2_INTENSITY-BASED_QuartileCoefficientOfDispersion | 0.963 (0.945 - 0.975) | <0.0001*** |
| T2_GLCM_JointEntropyLog10 | 0.963 (0.945 - 0.975) | <0.0001*** |
| T2_GLCM_JointEntropyLog2 | 0.963 (0.945 - 0.975) | <0.0001*** |
| T2_GLCM_DifferenceEntropy | 0.963 (0.945 - 0.975) | <0.0001*** |
| T2_GLSZM_SmallZoneEmphasis | 0.963 (0.945 - 0.975) | <0.0001*** |
| ADC_INTENSITY-HISTOGRAM_INTENSITYHistogramMedianAbsoluteDeviation | 0.963 (0.944 - 0.975) | <0.0001*** |
| ADC_INTENSITY-BASED_MedianAbsoluteDeviation | 0.963 (0.944 - 0.975) | <0.0001*** |
| T2_NGTDM_Contrast | 0.962 (0.944 - 0.975) | <0.0001*** |
| ADC_INTENSITY-HISTOGRAM_INTENSITYHistogramMeanAbsoluteDeviation | 0.962 (0.943 - 0.975) | <0.0001*** |
| T2_GLCM4_ClusterProminence | 0.962 (0.944 - 0.975) | <0.0001*** |
| ADC_INTENSITY-BASED_MeanAbsoluteDeviation | 0.962 (0.943 - 0.975) | <0.0001*** |
| T2_GLCM_SumEntropy | 0.962 (0.943 - 0.974) | <0.0001*** |
| T2_INTENSITY-HISTOGRAM_INTENSITYHistogramInterquartileRange | 0.961 (0.941 - 0.974) | <0.0001*** |
| T2_GLCM2_JointEntropyLog2 | 0.96 (0.941 - 0.974) | <0.0001*** |
| T2_GLCM2_DifferenceEntropy | 0.96 (0.941 - 0.974) | <0.0001*** |
| T2_GLCM2_JointEntropyLog10 | 0.96 (0.941 - 0.974) | <0.0001*** |
| T2_GLCM2_SumEntropy | 0.96 (0.94 - 0.973) | <0.0001*** |
| ADC_INTENSITY-HISTOGRAM_INTENSITYHistogramMaximumGreyLevel | 0.959 (0.938 - 0.973) | <0.0001*** |
| ADC_GLCM2_InverseVariance | 0.959 (0.938 - 0.973) | <0.0001*** |
| ADC_GLCM_InverseVariance | 0.959 (0.938 - 0.973) | <0.0001*** |
| T2_GLCM_AngularSecondMoment | 0.959 (0.938 - 0.972) | <0.0001*** |
| T2_INTENSITY-HISTOGRAM_INTENSITYHistogramStandardDeviation | 0.959 (0.938 - 0.972) | <0.0001*** |
| T2_INTENSITY-BASED_StandardDeviation | 0.959 (0.938 - 0.972) | <0.0001*** |
| ADC_INTENSITY-BASED_MaximumGreyLevel | 0.957 (0.935 - 0.971) | <0.0001*** |
| T2_GLSZM_GreyLevelVariance | 0.957 (0.936 - 0.971) | <0.0001*** |
| T2_GLCM2_JointMaximum | 0.957 (0.935 - 0.971) | <0.0001*** |
| T2_GLCM4_JointEntropyLog10 | 0.956 (0.935 - 0.971) | <0.0001*** |
| T2_GLCM4_JointEntropyLog2 | 0.956 (0.935 - 0.971) | <0.0001*** |
| T2_GLCM4_DifferenceEntropy | 0.956 (0.935 - 0.971) | <0.0001*** |
| T2_GLCM4_SumEntropy | 0.956 (0.934 - 0.97) | <0.0001*** |
| T2_GLCM4_AngularSecondMoment | 0.954 (0.932 - 0.969) | <0.0001*** |
| T2_GLCM2_AngularSecondMoment | 0.954 (0.932 - 0.969) | <0.0001*** |
| ADC_GLSZM_SmallZoneLowGreyLevelEmphasis | 0.954 (0.931 - 0.97) | <0.0001*** |
| ADC_GLRLM_ShortRunLowGreyLevelEmphasis | 0.95 (0.925 - 0.967) | <0.0001*** |
| ADC_GLSZM_ZoneSizeVariance | 0.95 (0.925 - 0.967) | <0.0001*** |
| ADC_INTENSITY-HISTOGRAM_INTENSITYHistogramCoefficientOfVariation | 0.947 (0.921 - 0.965) | <0.0001*** |

| | | |
|---|---|---|
| ADC_INTENSITY-HISTOGRAM_INTENSITYHistogramStandardDeviation | 0.947 (0.92 - 0.965) | <0.0001*** |
| ADC_INTENSITY-BASED_StandardDeviation | 0.947 (0.92 - 0.965) | <0.0001*** |
| T2_MORPHOLOGICAL_IntegratedT2_INTENSITY | 0.946 (0.92 - 0.964) | <0.0001*** |
| T2_INTENSITY-HISTOGRAM_INTENSITYHistogramQuartileCoefficientOfDispersion | 0.946 (0.92 - 0.964) | <0.0001*** |
| T2_MORPHOLOGICAL_Compacity | 0.943 (0.915 - 0.962) | <0.0001*** |
| T2_INTENSITY-HISTOGRAM_UniformityIBSI | 0.942 (0.914 - 0.961) | <0.0001*** |
| ADC_INTENSITY-HISTOGRAM_MaximumHistogramGradient | 0.941 (0.911 - 0.961) | <0.0001*** |
| ADC_INTENSITY-HISTOGRAM_MinimumHistogramGradient | 0.938 (0.907 - 0.959) | <0.0001*** |
| T2_INTENSITY-HISTOGRAM_INTENSITYHistogramEntropyLog2 | 0.937 (0.906 - 0.957) | <0.0001*** |
| T2_INTENSITY-HISTOGRAM_INTENSITYHistogramEntropyLog10 | 0.937 (0.906 - 0.957) | <0.0001*** |
| T2_INTENSITY-HISTOGRAM_INTENSITYHistogramCoefficientOfVariation | 0.936 (0.905 - 0.957) | <0.0001*** |
| T2_INTENSITY-BASED_CoefficientOfVariation | 0.936 (0.905 - 0.957) | <0.0001*** |
| T2_NGTDM_Complexity | 0.935 (0.904 - 0.956) | <0.0001*** |
| ADC_NGTDM_Busyness | 0.935 (0.899 - 0.958) | <0.0001*** |
| ADC_GLCM_Contrast | 0.934 (0.901 - 0.956) | <0.0001*** |
| T2_MORPHOLOGICAL_Volume | 0.933 (0.9 - 0.955) | <0.0001*** |
| ADC_INTENSITY-BASED_QuartileCoefficientOfDispersion | 0.93 (0.895 - 0.953) | <0.0001*** |
| ADC_NGTDM_Strength | 0.929 (0.894 - 0.953) | <0.0001*** |
| T2_INTENSITY-HISTOGRAM_MaximumHistogramGradient | 0.928 (0.894 - 0.951) | <0.0001*** |
| T2_INTENSITY-BASED_Energy | 0.924 (0.889 - 0.949) | <0.0001*** |
| T2_GLCM_Correlation | 0.924 (0.888 - 0.949) | <0.0001*** |
| ADC_GLCM2_NormalisedInverseDifference | 0.923 (0.885 - 0.949) | <0.0001*** |
| T2_MORPHOLOGICAL_Maximum3DDiameter | 0.919 (0.881 - 0.945) | <0.0001*** |
| T2_MORPHOLOGICAL_SurfaceToVolumeRatio | 0.918 (0.88 - 0.945) | <0.0001*** |
| T2_MORPHOLOGICAL_Sphericity | 0.918 (0.879 - 0.945) | <0.0001*** |
| ADC_GLRLM_GreyLevelNonUniformity | 0.918 (0.878 - 0.945) | <0.0001*** |
| ADC_GLCM_NormalisedInverseDifference | 0.918 (0.877 - 0.945) | <0.0001*** |
| T2_MORPHOLOGICAL_SphericalDisproportion | 0.917 (0.878 - 0.944) | <0.0001*** |
| T2_MORPHOLOGICAL_Asphericity | 0.917 (0.878 - 0.944) | <0.0001*** |
| ADC_INTENSITY-BASED_Energy | 0.916 (0.874 - 0.944) | <0.0001*** |
| T2_MORPHOLOGICAL_Compactness1 | 0.915 (0.876 - 0.943) | <0.0001*** |
| ADC_GLCM2_Contrast | 0.915 (0.873 - 0.944) | <0.0001*** |
| T2_INTENSITY-HISTOGRAM_MinimumHistogramGradient | 0.913 (0.872 - 0.941) | <0.0001*** |
| ADC_GLCM4_SumVariance | 0.912 (0.869 - 0.941) | <0.0001*** |
| ADC_GLCM4_ClusterTendency | 0.912 (0.869 - 0.941) | <0.0001*** |
| ADC_NGTDM_Contrast | 0.909 (0.86 - 0.941) | <0.0001*** |
| T2_MORPHOLOGICAL_SurfaceArea | 0.908 (0.866 - 0.938) | <0.0001*** |
| ADC_GLSZM_GreyLevelNonUniformity | 0.908 (0.863 - 0.939) | <0.0001*** |
| T2_MORPHOLOGICAL_Compactness2 | 0.904 (0.859 - 0.935) | <0.0001*** |
| T2_GLCM2_Correlation | 0.9 (0.853 - 0.932) | <0.0001*** |
| T2_GLRLM_GreyLevelNonUniformity | 0.895 (0.847 - 0.929) | <0.0001*** |
| ADC_GLCM_DifferenceVariance | 0.894 (0.843 - 0.929) | <0.0001*** |
| ADC_GLCM2_JointVariance | 0.892 (0.84 - 0.928) | <0.0001*** |
| ADC_GLCM_JointVariance | 0.889 (0.835 - 0.925) | <0.0001*** |
| ADC_INTENSITY-HISTOGRAM_INTENSITYHistogramRange | 0.888 (0.834 - 0.925) | <0.0001*** |
| ADC_INTENSITY-BASED_MinimumGreyLevel | 0.887 (0.832 - 0.924) | <0.0001*** |
| T2_GLSZM_GreyLevelNonUniformity | 0.885 (0.831 - 0.922) | <0.0001*** |
| ADC_INTENSITY-HISTOGRAM_INTENSITYHistogramMinimumGreyLevel | 0.884 (0.829 - 0.922) | <0.0001*** |
| ADC_INTENSITY-BASED_Range | 0.883 (0.826 - 0.921) | <0.0001*** |
| ADC_GLCM4_InverseVariance | 0.881 (0.824 - 0.921) | <0.0001*** |
| T2_GLCM4_NormalisedInverseDifference | 0.879 (0.824 - 0.918) | <0.0001*** |
| ADC_INTENSITY-BASED_CoefficientOfVariation | 0.879 (0.821 - 0.919) | <0.0001*** |
| T2_GLCM4_NormalisedInverseDifferenceMoment | 0.878 (0.822 - 0.917) | <0.0001*** |
| T2_GLCM2_NormalisedInverseDifference | 0.878 (0.822 - 0.917) | <0.0001*** |
| T2_GLCM2_NormalisedInverseDifferenceMoment | 0.877 (0.82 - 0.916) | <0.0001*** |
| T2_GLSZM_NormalisedGreyLevelNonUniformity | 0.877 (0.82 - 0.916) | <0.0001*** |
| ADC_GLRLM_RunLengthNonUniformity | 0.875 (0.816 - 0.916) | <0.0001*** |
| ADC_GLSZM_ZoneSizeNonUniformity | 0.874 (0.813 - 0.915) | <0.0001*** |
| ADC_GLCM4_JointVariance | 0.872 (0.811 - 0.914) | <0.0001*** |
| ADC_GLCM2_DifferenceVariance | 0.871 (0.809 - 0.913) | <0.0001*** |
| T2_MORPHOLOGICAL_CentreOfMassShift | 0.871 (0.811 - 0.912) | <0.0001*** |
| T2_NGTDM_Busyness | 0.87 (0.81 - 0.911) | <0.0001*** |
| T2_GLCM_NormalisedInverseDifference | 0.868 (0.808 - 0.91) | <0.0001*** |
| T2_GLSZM_ZoneSizeNonUniformity | 0.866 (0.806 - 0.909) | <0.0001*** |
| T2_INTENSITY-BASED_Skewness | 0.863 (0.801 - 0.907) | <0.0001*** |
| T2_INTENSITY-HISTOGRAM_INTENSITYHistogramSkewness | 0.862 (0.8 - 0.906) | <0.0001*** |
| T2_GLCM_NormalisedInverseDifferenceMoment | 0.859 (0.796 - 0.904) | <0.0001*** |
| T2_NGTDM_Strength | 0.859 (0.796 - 0.904) | <0.0001*** |
| ADC_INTENSITY-HISTOGRAM_INTENSITYHistogramVariance | 0.851 (0.782 - 0.9) | <0.0001*** |

| | | |
|---|---|---|
| ADC_INTENSITY-BASED_Variance | 0.851 (0.781 - 0.9) | <0.0001*** |
| T2_GLCM4_Correlation | 0.848 (0.78 - 0.897) | <0.0001*** |
| T2_GLRLM_RunLengthNonUniformity | 0.84 (0.769 - 0.891) | <0.0001*** |
| T2_MORPHOLOGICAL_ApproximateVolume | 0.838 (0.766 - 0.889) | <0.0001*** |
| T2_MORPHOLOGICAL_voxelsCounting | 0.838 (0.766 - 0.889) | <0.0001*** |
| ADC_GLSZM_GreyLevelVariance | 0.835 (0.759 - 0.889) | <0.0001*** |
| T2_INTENSITY-BASED_Kurtosis | 0.831 (0.757 - 0.885) | <0.0001*** |
| T2_INTENSITY-HISTOGRAM_INTENSITYHistogramKurtosis | 0.83 (0.755 - 0.883) | <0.0001*** |
| T2_INTENSITY-BASED_MaximumGreyLevel | 0.824 (0.747 - 0.88) | <0.0001*** |
| T2_INTENSITY-HISTOGRAM_INTENSITYHistogramMaximumGreyLevel | 0.823 (0.746 - 0.879) | <0.0001*** |
| T2_GLSZM_LowGrayLevelZoneEmphasis | 0.823 (0.745 - 0.879) | <0.0001*** |
| ADC_GLCM2_SumVariance | 0.82 (0.738 - 0.878) | <0.0001*** |
| ADC_GLCM2_ClusterTendency | 0.82 (0.738 - 0.878) | <0.0001*** |
| T2_GLRLM_LongRunLowGreyLevelEmphasis | 0.82 (0.741 - 0.876) | <0.0001*** |
| T2_GLRLM_LowGreyLevelRunEmphasis | 0.815 (0.734 - 0.873) | <0.0001*** |
| T2_GLSZM_SmallZoneLowGreyLevelEmphasis | 0.814 (0.733 - 0.873) | <0.0001*** |
| T2_GLRLM_ShortRunLowGreyLevelEmphasis | 0.813 (0.731 - 0.871) | <0.0001*** |
| ADC_INTENSITY-BASED_Skewness | 0.81 (0.725 - 0.871) | <0.0001*** |
| ADC_GLCM_SumVariance | 0.809 (0.723 - 0.871) | <0.0001*** |
| ADC_GLCM_ClusterTendency | 0.809 (0.723 - 0.871) | <0.0001*** |
| ADC_GLCM4_ClusterProminence | 0.808 (0.722 - 0.87) | <0.0001*** |
| ADC_GLSZM_LargeZoneLowGreyLevelEmphasis | 0.807 (0.721 - 0.869) | <0.0001*** |
| ADC_GLSZM_LargeZoneEmphasis | 0.807 (0.721 - 0.869) | <0.0001*** |
| ADC_GLSZM_LargeZoneHighGreyLevelEmphasis | 0.804 (0.716 - 0.867) | <0.0001*** |
| ADC_INTENSITY-HISTOGRAM_INTENSITYHistogramSkewness | 0.797 (0.699 - 0.866) | <0.0001*** |
| T2_INTENSITY-BASED_Range | 0.785 (0.694 - 0.852) | <0.0001*** |
| T2_INTENSITY-HISTOGRAM_INTENSITYHistogramRange | 0.785 (0.694 - 0.852) | <0.0001*** |
| ADC_GLCM2_NormalisedInverseDifferenceMoment | 0.782 (0.686 - 0.852) | <0.0001*** |
| ADC_NGTDM_Coarseness | 0.782 (0.677 - 0.855) | <0.0001*** |
| ADC_GLCM4_NormalisedInverseDifference | 0.779 (0.681 - 0.849) | <0.0001*** |
| T2_INTENSITY-HISTOGRAM_RootMeanSquare | 0.773 (0.677 - 0.843) | <0.0001*** |
| ADC_GLCM_SumEntropy | 0.771 (0.67 - 0.844) | <0.0001*** |
| ADC_GLCM2_SumEntropy | 0.766 (0.664 - 0.84) | <0.0001*** |
| ADC_GLCM_NormalisedInverseDifferenceMoment | 0.758 (0.653 - 0.835) | <0.0001*** |
| ADC_GLCM4_SumEntropy | 0.753 (0.647 - 0.831) | <0.0001*** |
| T2_NGTDM_Coarseness | 0.74 (0.634 - 0.82) | <0.0001*** |
| ADC_NGTDM_Complexity | 0.725 (0.609 - 0.811) | <0.0001*** |
| T2_GLSZM_ZoneSizeEntropy | 0.723 (0.61 - 0.806) | <0.0001*** |
| T2_INTENSITY-BASED_MinimumGreyLevel | 0.694 (0.573 - 0.786) | <0.0001*** |
| T2_INTENSITY-HISTOGRAM_INTENSITYHistogramMinimumGreyLevel | 0.691 (0.569 - 0.783) | <0.0001*** |
| ADC_GLCM4_DifferenceAverage | 0.63 (0.486 - 0.741) | <0.0001*** |
| ADC_GLCM4_Dissimilarity | 0.63 (0.486 - 0.741) | <0.0001*** |
| ADC_INTENSITY-BASED_Kurtosis | 0.613 (0.464 - 0.728) | <0.0001*** |
| ADC_INTENSITY-HISTOGRAM_INTENSITYHistogramKurtosis | 0.603 (0.439 - 0.727) | <0.0001*** |
| ADC_GLCM_ClusterShade | 0.603 (0.451 - 0.72) | <0.0001*** |
| ADC_GLCM4_ClusterShade | 0.593 (0.44 - 0.713) | <0.0001*** |
| ADC_GLCM2_ClusterShade | 0.577 (0.42 - 0.701) | <0.0001*** |
| ADC_GLCM_ClusterProminence | 0.549 (0.385 - 0.679) | <0.0001*** |
| ADC_GLCM2_ClusterProminence | 0.518 (0.348 - 0.656) | <0.0001*** |
| T2_GLCM4_InverseVariance | 0.45 (0.274 - 0.597) | <0.0001*** |
| T2_GLCM2_InverseVariance | 0.434 (0.256 - 0.585) | <0.0001*** |
| ADC_GLCM4_NormalisedInverseDifferenceMoment | 0.428 (0.242 - 0.584) | <0.0001*** |
| ADC_GLCM4_DifferenceVariance | 0.41 (0.221 - 0.569) | <0.0001*** |
| T2_GLCM_InverseVariance | 0.405 (0.222 - 0.561) | <0.0001*** |
| ADC_GLCM4_Contrast | 0.372 (0.178 - 0.538) | 0,0002*** |
| ADC_GLCM4_Correlation | 0.158 (-0.051 - 0.354) | 0,0688 |

NOTE.- ***: *P*<0.0001

**Supplementary Table S3.** Understanding unsupervised radiomics clusters

| Characteristics | Cluster T2 | | adjusted *P*-value | Cluster ADC | | | adjusted *P*-value | Cluster T2+ADC | | adjusted *P*-value |
|---|---|---|---|---|---|---|---|---|---|---|
| | Group T2-1 | Group T2-2 | | Group ADC-1 | Group ADC-2 | Group ADC-3 | | Group T2+ADC-1 | Group T2+ADC-2 | |
| **Initial prostate volume** | 89.6 ± 38.6 | 99.8 ± 43.2 | 0.3104 | 85.1 ± 40.6 | 96.6 ± 35.1 | 97.2 ± 44.4 | 0.4560 | 86.4 ± 39.1 | 99.9 ± 41.8 | 0.1866 |
| **Initial IPSS** | 19.2 ± 6.1 | 18.5 ± 6.1 | 0.5936 | 19.7 ± 4.63 | 17.5 ± 6.86 | 19.2 ± 6.07 | 0.4960 | 19.3 ± 6.5 | 18.6 ± 5.8 | 0.6562 |
| **Initial QOL** | 4.8 ± 1.3 | 4.7 ± 1.2 | 0.8714 | 4.8 ± 1.4 | 4.7 ± 1.0 | 4.74 ± 1.2 | 0.807 | 4.6 ± 1.2 | 4.8 ± 0.8 | 0.4558 |
| **Wasserman classification** | | | 0.2170 | | | | 0.2274 | 12/37 (32.4) | | 0.2170 |
| Type 1 | 17/35 (48.6) | 12/37 (32.4) | | 8/13 (61.5) | 6/20 (30) | 15/39 (38.5) | | 14/27 (51.9) | 15/45 (33.3) | |
| Type 2 | 7/35 (20) | 4/37 (10.8) | | 2/13 (15.4) | 5/20 (25) | 4/39 (10.3) | | 5/27 (18.5) | 6/45 (13.3) | |
| Type 3 | 11/35 (31.4) | 21/37 (56.8) | | 3/13 (23.1) | 9/20 (45) | 20/39 (51.3) | | 8/27 (29.6) | 24/45 (53.3) | |
| **adBPH** | | | **0.0291*** | | | | 0.3290 | | | 0.2363 |
| No | 21/35 (60) | 10/37 (27) | | 8/13 (61.5) | 8/20 (40) | 15/39 (38.5) | | 15/27 (55.6) | 16/45 (35.6) | |
| Yes | 14/35 (40) | 27/37 (73) | | 5/13 (38.5) | 12/20 (60) | 24/39 (61.5) | | 12/27 (44.4) | 29/45 (64.4) | |
| **Clinical success at M3** | | | 0.8590 | | | | 1 | | | 0.8590 |
| No | 6/35 (17.1) | 7/37 (18.9) | | 2/13 (15.4) | 4/20 (20) | 7/39 (17.9) | | 6/27 (22.2) | 7/45 (15.6) | |
| Yes | 29/35 (82.9) | 30/37 (81.1) | | 11/13 (84.6) | 16/20 (80) | 32/39 (82.1) | | 21/27 (77.8) | 38/45 (84.4) | |
| **Clinical success at Y2** | | | 1 | | | | 1 | | | 1 |
| No | 14/35 (40) | 15/37 (40.5) | | 5/13 (38.5) | 10/20 (50) | 14/39 (35.9) | | 13/27 (48.1) | 16/45 (35.6) | |
| Yes | 21/35 (60) | 22/37 (59.5) | | 8/13 (61.5) | 10/20 (50) | 25/39 (64.1) | | 14/27 (51.9) | 29/45 (64.4) | |

NOTE.- *: adjusted $P<0.05$ (according to Benjamini-Hochberg procedure). All tests were non-parametric tests, either unpaired Mann-Whitney test, or Kruskal-Wallis test, or Chi-square test.

For continuous variables (volume, IPSS and QOL) data are mean ± standard deviation. For categorical, they are number of patients with percentage in parentheses.

Abbreviations: adBPH: adenomatous dominant benign prostate hypertrophy, ADC: apparent diffusion coefficient, IPSS: international prostate score symptom M3: at 3 months after prostate artery embolization, QOL: quality of life, Y2: at 2 years after prostate artery embolization.

**Supplementary Table S4.** Radiomics features selected by Elasticnet

| Selected radiomics features | Coefficients |
|---|---|
| T2_GLSZM_ZoneSizeVariance | 5.655 |
| ADC_GLCM_InverseVariance | 5.644 |
| ADC_GLCM_Correlation | 5.307 |
| T2_MORPHOLOGICAL_SurfaceToVolumeRatio | 4.087 |
| ADC_GLCM_NormalisedInverseDifference | 3.428 |
| T2_MORPHOLOGICAL_Volume | 3.859 |
| T2_INTENSITY-BASED_Skewness | 2.687 |
| ADC_INTENSITY-HISTOGRAM_INTENSITYHistogram10thPercentile | 2.346 |
| ADC_INTENSITY-HISTOGRAM_INTENSITYHistogramMinimumGreyLevel | 2.149 |
| ADC_INTENSITY-HISTOGRAM_INTENSITYHistogramQuartileCoefficientOfDispersion | 1.435 |
| T2_INTENSITY-BASED_50thPercentile | 0.037 |
| T2_GLSZM_LargeZoneLowGreyLevelEmphasis | -0.056 |
| T2_MORPHOLOGICAL_CentreOfMassShift | -0.225 |
| T2_MORPHOLOGICAL_Compactness2 | -0.275 |
| T2_GLCM_NormalisedInverseDifferenceMoment | -0.283 |
| ADC_GLSZM_ZoneSizeVariance | -0.510 |
| T2_GLCM2_ClusterShade | -0.695 |
| T2_NGTDM_Busyness | -1.229 |
| T2_NGTDM_Strength | -1.280 |
| T2_GLCM2_Correlation | -1.329 |
| ADC_NGTDM_Strength | -1.369 |
| T2_INTENSITY-HISTOGRAM_MaximumHistogramGradientGreyLevel | -1.467 |

NOTE.- The selected radiomics features are ordered in descending order according to their coefficient (i.e., strength in the final model)
